# Machine Learning Analysis of Sex Differences in Cardiovascular-Kidney-Metabolic Risk Factors and Prognosis Among Patients With Moderate-to-Severe Coronary Artery Calcification: Prospective Cohort Study

**DOI:** 10.2196/82742

**Published:** 2026-07-16

**Authors:** Zixiang Ye, Enmin Xie, Zhangyu Lin, Chenxi Song, Rui Zhang, Hao-Yu Wang, Shanshan Shi, Zhiyong Zhao, Hao Wang, Lei Feng, Kefei Dou

**Affiliations:** 1 Department of Cardiology Fuwai Hospital Chinese Academy of Medical Sciences and Peking Union Medical College Beijing China; 2 State Key Laboratory of Cardiovascular Disease Beijing China; 3 Cardiometabolic Medicine Center Fuwai Hospital Chinese Academy of Medical Sciences and Peking Union Medical College Beijing China

**Keywords:** sex difference, coronary artery calcification, cardiovascular-kidney-metabolic risk factors, extreme gradient boosting, prognosis

## Abstract

**Background:**

Patients with coronary artery calcification exhibit notable sex differences in clinical presentation, particularly concerning the role of cardiovascular-kidney-metabolic (CKM) risk factors and their impact on prognoses. However, the precise nature of these sex-specific differences remains incompletely understood.

**Objective:**

This study aimed to investigate sex disparities in CKM risk factors among patients with moderate-to-severe coronary artery calcification (MSCAC) and elucidate their association with adverse clinical outcomes.

**Methods:**

A total of 2418 patients with MSCAC undergoing their first percutaneous coronary intervention were included. Hazard ratios (HRs) were computed to evaluate sex differences in the prognostic significance of various CKM risk factors, including chronic kidney disease (CKD), diabetes mellitus (DM), obesity, hypertension, and hypertriglyceridemia. Four machine learning models—logistic regression, extreme gradient boosting (XGBoost), random forest, and support vector machine—were constructed to predict adverse events. The best-performing model was interpreted using Shapley additive explanations (SHAP) values to identify the relative importance of CKM risk factors and clarify potential sex disparities. Major adverse cardiovascular events (MACEs) were defined as all-cause mortality, nonfatal myocardial infarction, and unplanned repeat revascularization.

**Results:**

Among the participants, 86.9% (2101/2418) had ≥1 CKM risk factor, while 3.1% (74/2418) had ≥4 risk factors. CKD was independently associated with the occurrence of MACEs in both female patients (HR 2.65, 95% CI 1.50-4.69) and male patients (HR 1.53, 95% CI 1.02-2.60). Notably, the association was stronger in female patients, with a female-to-male multivariate-adjusted HR ratio for CKD of 1.68 (95% CI 1.04-2.97). DM was also associated with MACEs in both sexes, with adjusted HRs of 1.20 (95% CI 1.02-1.92) in female patients and 1.59 (95% CI 1.19-2.12) in male patients. Among the models evaluated, XGBoost demonstrated the highest predictive performance in the test set (area under the curve 0.92; average precision 0.92; *F*_1_-score=0.86). XGBoost maintained good predictive performance in the external validation cohort (area under the curve 0.86; average precision 0.71; *F*_1_-score=0.68). Baseline DM was identified as the CKM risk factor with the highest feature importance for MACEs, with SHAP values of 0.17 in female patients and 0.23 in male patients. Conversely, CKD emerged as the most important CKM risk factor in female patients (SHAP value 0.18), while DM ranked highest in male participants.

**Conclusions:**

This study confirmed that CKM risk factors and their influence on prognosis in patients with MSCAC exhibit significant sex differences. The application of machine learning, particularly XGBoost, facilitates a deeper understanding of these disparities and provides a basis for personalized, sex-specific risk assessment and targeted interventions for patients with MSCAC.

## Introduction

Moderate-to-severe coronary artery calcification (MSCAC) is a high-risk, complex form of coronary artery disease (CAD). Patients with MSCAC often exhibit irreversible vascular calcification, which complicates percutaneous coronary intervention (PCI) procedures and is associated with a poorer prognosis [1,2]. Therefore, the early identification of high-risk factors in these patients is of crucial importance. Metabolic disturbances and electrolyte imbalances are key contributors to the development of severe coronary artery calcification (CAC). Dysregulated lipid deposition in the coronary vessel wall can lead to unstable plaques, while disturbances in calcium-phosphorus metabolism further exacerbate vascular calcification [3-6]. Consequently, cardiovascular metabolic factors, including renal function, are significantly associated with the occurrence of severe CAC.

This perspective aligns with the concept of cardiovascular-kidney-metabolic (CKM) health emphasized by the American Heart Association (AHA), which highlights the importance of multiorgan management, particularly of cardiovascular, metabolic, and renal conditions [7,8]. According to AHA reports, multiple risk factors can contribute to the development and progression of CKM disorders. These include chronic kidney disease (CKD), hypertension, diabetes mellitus (DM), hypertriglyceridemia (HTG), and obesity, all of which promote excessive lipid accumulation, immune inflammation, oxidative stress, and insulin resistance, thereby further accelerating vascular calcification [9,10]. Effective management of CKM risk factors is essential for reducing adverse cardiovascular events among patients with MSCAC.

However, the incidence of CAC varies by sex, and the impact of CKM risk factors may also differ between male patients and female patients. Limited research has explored the sex-specific effects of these risk factors on adverse outcomes in patients with MSCAC.

On the basis of this background, the primary aims of this study were (1) to assess the distribution of CKM risk factors—including CKD, DM, obesity, hypertension, and HTG—among male and female patients with MSCAC following their first PCI; (2) to establish machine learning (ML) models predicting adverse prognosis in patients with MSCAC based on these CKM risk factors; and (3) to explore potential sex differences in the impact of these CKM risk factors on prognosis using the ML models.

## Methods

### Ethical Considerations

The study was approved by the ethics committee of Fuwai Hospital (2016-847) and complied with the principles of the Declaration of Helsinki. The enrolled patients provided informed consent for their treatment. Participants were assured that their privacy and confidentiality would be strictly protected, and all data were anonymized prior to analysis. No personal identifying information was collected or stored.

### Study Population

**Figure 1:**
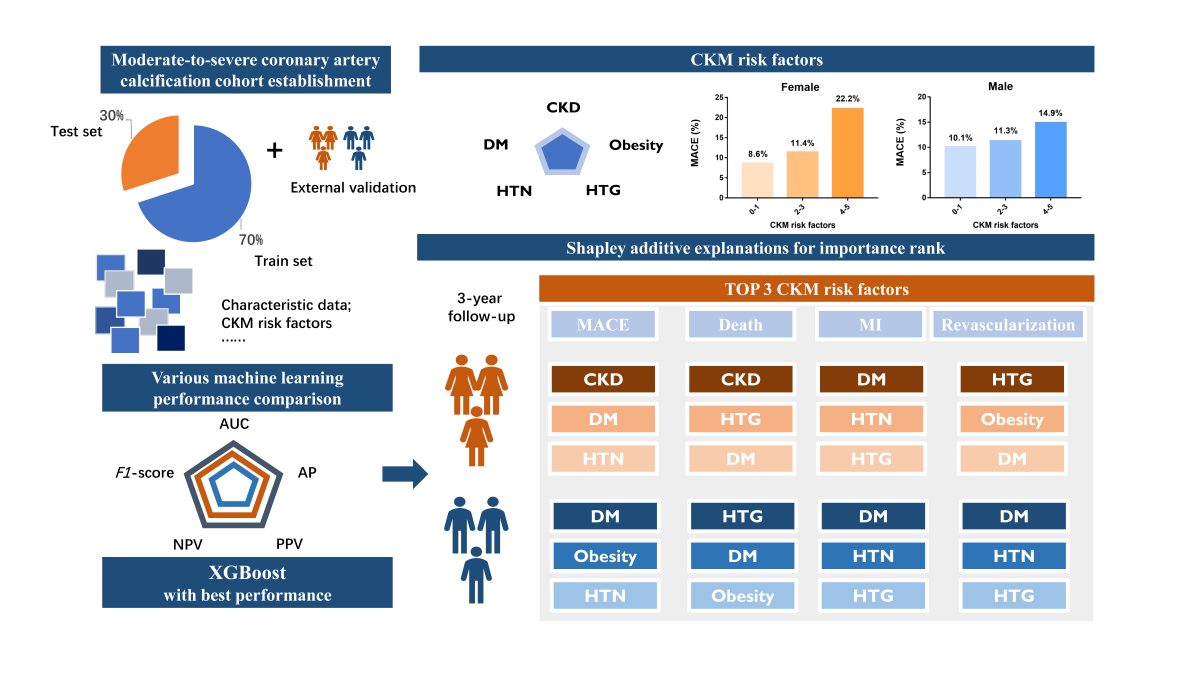
Overview of the study design and workflow. AP: average precision; AUC: area under the curve; CKD: chronic kidney disease; CKM: cardiovascular-kidney-metabolic; DM: diabetes mellitus; HTG: hypertriglyceridemia; HTN: hypertension; MACE: major adverse cardiovascular events; MI: myocardial infarction; NPV: negative predictive value; PPV: positive predictive value; XGBoost: extreme gradient boosting.

This study was a retrospective analysis of a prospective cohort conducted at Fuwai Hospital. The cohort consisted of patients with MSCAC who underwent their first PCI between January 2017 and December 2018. All participants provided informed consent. An external validation cohort, which included patients with MSCAC who underwent their first PCI from January 2021 to June 2021, was conducted to validate the predictive performance of the ML model as well. The study design is illustrated in Figure 1.

Inclusion criteria were (1) a diagnosis of CAD and receipt of PCI for the first time at this institution and (2) a diagnosis of MSCAC based on angiography. Exclusion criteria included the following: prior PCI or coronary artery bypass grafting, missing baseline serum creatinine or triglyceride data, and in-hospital death. The detailed inclusion and exclusion criteria for the development and external validation sets are summarized in Figures S1 and S2 in Multimedia Appendix 1. All participants were followed up regularly by trained investigators after discharge.

### CKM Risk Factors

In this study, we investigated 5 CKM risk factors following the AHA Presidential Advisory, including CKD, DM, obesity, hypertension, and HTG. Estimated glomerular filtration rate (eGFR) was calculated using the CKD-EPI equation based on the first serum creatinine measurement at admission [11]. CKD was defined as chronic renal disease or eGFR <60 mL/min/1.73 m^2^. DM was diagnosed as hemoglobin A_1c_ (HbA_1c_) of ≥6.5%, fasting plasma glucose levels of ≥7.0 mmol/L, or current use of insulin or antihyperglycemic medications. Obesity was defined as a BMI of ≥30 kg/m^2^. Hypertension was defined as systolic blood pressure of ≥140 mm Hg, diastolic blood pressure of ≥80 mm Hg, a history of hypertension, or the use of antihypertensive medications. HTG was defined as triglyceride levels of ≥150 mg/100 mL (1.69 mmol/L).

### Clinical Outcomes

The study’s primary end point was major adverse cardiovascular events (MACEs), defined as a composite of all-cause mortality, nonfatal myocardial infarction, and unplanned repeat revascularization. The definition of each component of MACEs is described in Table S1 in Multimedia Appendix 2.

### ML Models

Four ML algorithms for binary classification were established, including logistic regression, extreme gradient boosting (XGBoost), random forest, and support vector machine. These classifiers were chosen for their potential strong performance with imbalanced data categorization, scalability, and resilience. The predictor variables were selected based on the Boruta algorithm and clinical significance. Prior to the development and internal validation of the ML models, the dataset was randomly partitioned into a training set (70%) and an independent holdout test set (30%). To address class imbalance in the binary composite outcome, the synthetic minority oversampling technique (SMOTE) with sampling_strategy=‘auto’ was applied exclusively to the training set. SMOTE was not applied to the validation folds or the independent test set to prevent data leakage. Hyperparameter optimization was performed using an adaptive resampling technique. Full details of the hyperparameter search space and the best parameters selected for the final model are provided in Table S2 in Multimedia Appendix 2.

### Statistical Analysis

Patient characteristics are summarized using medians with IQRs for continuous variables and frequencies with percentages for categorical variables. Missing data in baseline covariates were visually explored using the Visualization and Imputation of Missing Values package (Figure S3 in Multimedia Appendix 1) and assessed with the Little missing completely at random test (*P*<.05), supporting the assumption of missing at random. Multiple imputation by chained equations was performed using the mice package.

Several performance metrics were calculated and derived from each set of ML models, including the area under the curve (AUC), average precision (AP), positive predictive value (PPV), negative predictive value (NPV), and *F*_1_-score. To quantify uncertainty, 95% CIs for all metrics were calculated using bootstrap resampling with 2000 iterations. Calibration was assessed both visually (using calibration plots) and quantitatively by calculating the calibration slope, intercept, and Brier score. Calibration curves were also used to evaluate the best-performing model. The best-performing ML model was selected for subsequent analyses. Decision curve analysis was conducted to evaluate its clinical utility by quantifying the net benefit at various threshold probabilities. The interpretation of the predictive model was facilitated by Shapley additive explanations (SHAP), a comprehensive methodology designed to accurately quantify the contribution and influence of each feature on the final predictions. The importance of CKM risk factors within the dataset was interpreted using their corresponding SHAP values.

Multivariable Cox proportional hazards models were further used to calculate hazard ratios (HRs) and 95% CIs for MACEs associated with each CKM risk factor stratified by sex. Restricted cubic spline analysis was performed for eGFR, HbA_1c_, BMI, triglyceride, and systolic blood pressure. All models were adjusted for age, smoking status, history of CAD, acute coronary syndrome, BMI, low-density lipoprotein cholesterol, number of stents, and the SYNTAX (synergy between PCI with TAXUS and cardiac surgery) score. To formally assess sex differences, the female-to-male ratio of HRs with 95% CIs was calculated for each CKM risk factor. In addition, the overall CKM burden was categorized into 3 levels (0-1, 2-3, and 4-5), and the interaction term (sex×CKM category) was tested in the multivariable Cox model.

A 2-sided *P* value <.05 was considered statistically significant. All statistical analyses and calculations were conducted using R software (version 4.1.3; R Foundation for Statistical Computing) and Python (version 3.9.12; Python Software Foundation).

## Results

### Characteristics of Patients With MSCAC

**Figure 2:**
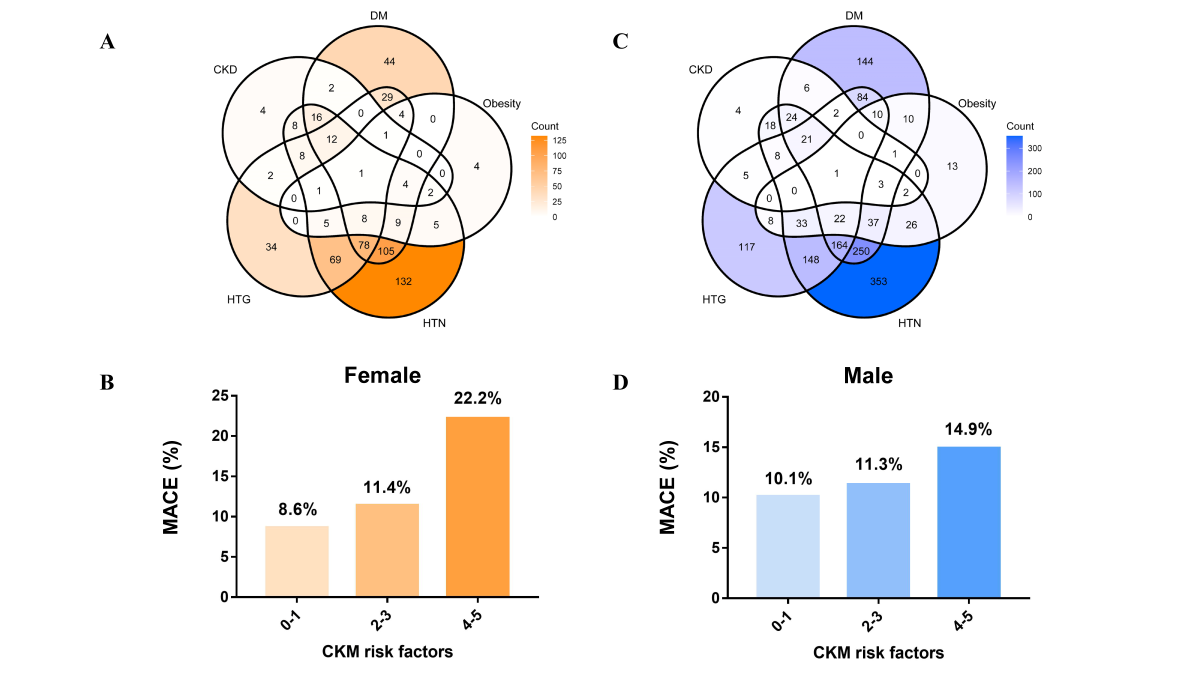
Distribution of cardiovascular-kidney-metabolic (CKM) risk factors and incidence of major adverse cardiovascular events (MACEs). (A) Venn diagram illustrating the distribution of CKM risk factors among female patients, (B) the chart of incidence rates of MACEs in female patients with varying numbers of CKM risk factors, (C) Venn diagram illustrating the distribution of CKM risk factors among male patients, and (D) the chart of incidence rates of MACEs in male patients with varying numbers of CKM risk factors. CKD: chronic kidney disease; DM: diabetes mellitus; HTG: hypertriglyceridemia; HTN: hypertension.

A total of 2418 patients with MSCAC were included in this study, of whom 647 (26.8%) were female. The median age was 62.4 (IQR 54.7-68.9) years. Regarding comorbidities, 156 (6.5%) patients had CKD, 1092 (45.2%) patients had DM, 210 (8.7%) patients were obese, 1573 (65.1%) patients had hypertension, and 875 (36.2%) patients had HTG. The distribution of the number of CKM risk factors—0, 1, 2, 3, 4, or 5—was 13.1%, 35.1%, 32.1%, 16.7%, 3%, and 0.1%, respectively. The prevalence of specific CKM risk factors among male and female patients, as well as in the overall cohort, is shown in Figure 2 and Figure S4 in Multimedia Appendix 1. Female patients with MSCAC exhibited higher rates of CKD and hypertension than male patients, and the overall number of CKM risk factors was also greater in female patients (Table 1). The median follow-up duration was 3.1 (IQR 2.9-3.2) years. During this period, 263 (10.9%) patients experienced MACEs, including 71 (11%) female patients and 192 (10.8%) male patients.

**Table 1:** Baseline characteristics of the participants.

Characteristics			All participants (N=2418)		Female participants (n=647)		Male participants (n=1771)		*P* value	
Age (years), median (IQR)			62.40 (54.70-68.90)		66.60 (61.00-73.25)		60.60 (53.30-67.00)		<.001	
**Smoking status, n (%)**										<.001
	Never	882 (36.5)		560 (86.6)		322 (18.2)				
	Former	824 (34.1)		53 (8.2)		771 (43.5)				
	Current	712 (29.4)		34 (5.3)		678 (38.3)				
History of coronary artery disease, n (%)			409 (16.9)		81 (12.5)		328 (18.5)		.001	
Acute coronary syndrome, n (%)			1522 (62.9)		418 (64.6)		1104 (62.3)		.33	
**Physical measures and biomarkers, median (IQR)**										
	BMI (kg/m^2^)	25.39 (23.44-27.62)		24.97 (22.80-27.24)		25.61 (23.82-27.68)		<.001		
	Systolic blood pressure (mm Hg)	130.00 (120.00-142.00)		131.00 (120.00-147.00)		130.00 (120.00-140.00)		<.001		
	Estimated glomerular filtration rate (mL/min/1.73 m^2^)	88.57 (76.33-98.39)		83.00 (71.58-93.69)		90.43 (78.90-99.88)		<.001		
	Hemoglobin A_1c_ (%)	6.10 (5.70-7.00)		6.20 (5.80-7.10)		6.00 (5.60-7.00)		<.001		
	Total cholesterol (mmol/L)	3.95 (3.34-4.69)		4.23 (3.62-5.02)		3.85 (3.25-4.57)		<.001		
	Triglycerides (mmol/L)	1.43 (1.06-2.01)		1.49 (1.13-2.09)		1.41 (1.03-1.98)		.002		
	Low-density lipoprotein cholesterol (mmol/L)	2.34 (1.82-2.96)		2.44 (1.97-3.19)		2.30 (1.76-2.89)		<.001		
	High-density lipoprotein cholesterol (mmol/L)	1.09 (0.92-1.30)		1.23 (1.03-1.45)		1.05 (0.89-1.23)		<.001		
**Coronary artery angiography and percutaneous intervention**										
	Heavy calcification, n (%)	513 (21.2)		154 (23.8)		359 (20.3)		.07		
	SYNTAX score, median (IQR)	14.00 (8.00-21.00)		14.00 (8.00-21.00)		14.00 (8.00-21.00)		.75		
**CKM^a^ risk factors, n (%)**										
	Chronic kidney disease	156 (6.5)		61 (9.4)		95 (5.4)		<.001		
	Diabetes mellitus	1092 (45.2)		313 (48.4)		779 (44.0)		.06		
	Obesity	210 (8.7)		44 (6.8)		166 (9.4)		.06		
	Hypertension	1573 (65.1)		463 (71.6)		1110 (62.7)		<.001		
	Hypertriglyceridemia	875 (36.2)		252 (38.9)		623 (35.2)		.10		
**Number of CKM risk factors, n (%)**										.003
	0	317 (13.1)		60 (9.3)		257 (14.5)				
	1	849 (35.1)		218 (33.7)		631 (35.6)				
	2	775 (32.1)		220 (34.0)		555 (31.3)				
	3	403 (16.7)		122 (18.9)		281 (15.9)				
	4	72 (3.0)		26 (4.0)		46 (2.6)				
	5	2 (0.1)		1 (0.2)		1 (0.1)				

^a^CKM: cardiovascular-kidney-metabolic.

### CKM Risk Factors and Poor Prognosis

Multivariate Cox regression analysis revealed that CKD was independently associated with the occurrence of MACEs in both female patients (HR 2.65, 95% CI 1.50-4.69) and male patients (HR 1.53, 95% CI 1.02-2.60). Notably, the association was more pronounced in female patients, with a multivariate-adjusted female-to-male HR ratio for CKD of 1.68 (95% CI 1.04-2.97; Table 2). Prevalent DM was also associated with MACEs in both sexes, with adjusted HRs of 1.20 (95% CI 1.02-1.92) in female patients and 1.59 (95% CI 1.19-2.12) in male patients. Hypertension was linked to adverse prognosis in female patients (HR 1.56, 95% CI 1.08-2.80), but not in male patients (HR 0.94, 95% CI 0.71-1.26).

**Table 2:** Adjusted hazard ratios (HRs) for major adverse cardiovascular events (MACEs) associated with cardiovascular-kidney-metabolic risk factors^a^.

MACE	Female participants, HR (95% CI)	Male participants, HR (95% CI)	Female-to-male ratio, HR (95% CI)
Chronic kidney disease	2.65 (1.50-4.69)	1.53 (1.02-2.60)	1.68 (1.04-2.97)
Diabetes mellitus	1.20 (1.02-1.92)	1.59 (1.19-2.12)	1.29 (0.87-1.88)
Obesity	1.45 (0.66-3.18)	1.03 (0.89-1.20)	0.76 (0.42-1.37)
Hypertension	1.56 (1.08-2.80)	0.94 (0.71-1.26)	1.13 (0.68-1.89)
Hypertriglyceridemia	1.01 (0.63-1.65)	1.08 (0.83-1.32)	0.96 (0.65-1.40)

^a^All models were adjusted for age, smoking status, history of coronary artery disease, acute coronary syndrome, BMI, low-density lipoprotein cholesterol, number of stents, and SYNTAX score.

Further analyses explored the associations between eGFR, BMI, systolic blood pressure, HbA_1c_, triglycerides, and MACEs, as well as with other adverse outcomes. In multivariate models, lower eGFR was significantly associated with increased risks of MACEs, all-cause mortality, myocardial infarction, and unplanned repeat revascularization in both sexes. The relationship between HbA_1c_ and outcomes was U-shaped for MACEs and mortality but showed a positive correlation with myocardial infarction and revascularization (Figures S5-S8 in Multimedia Appendix 1).

**Figure 3:**
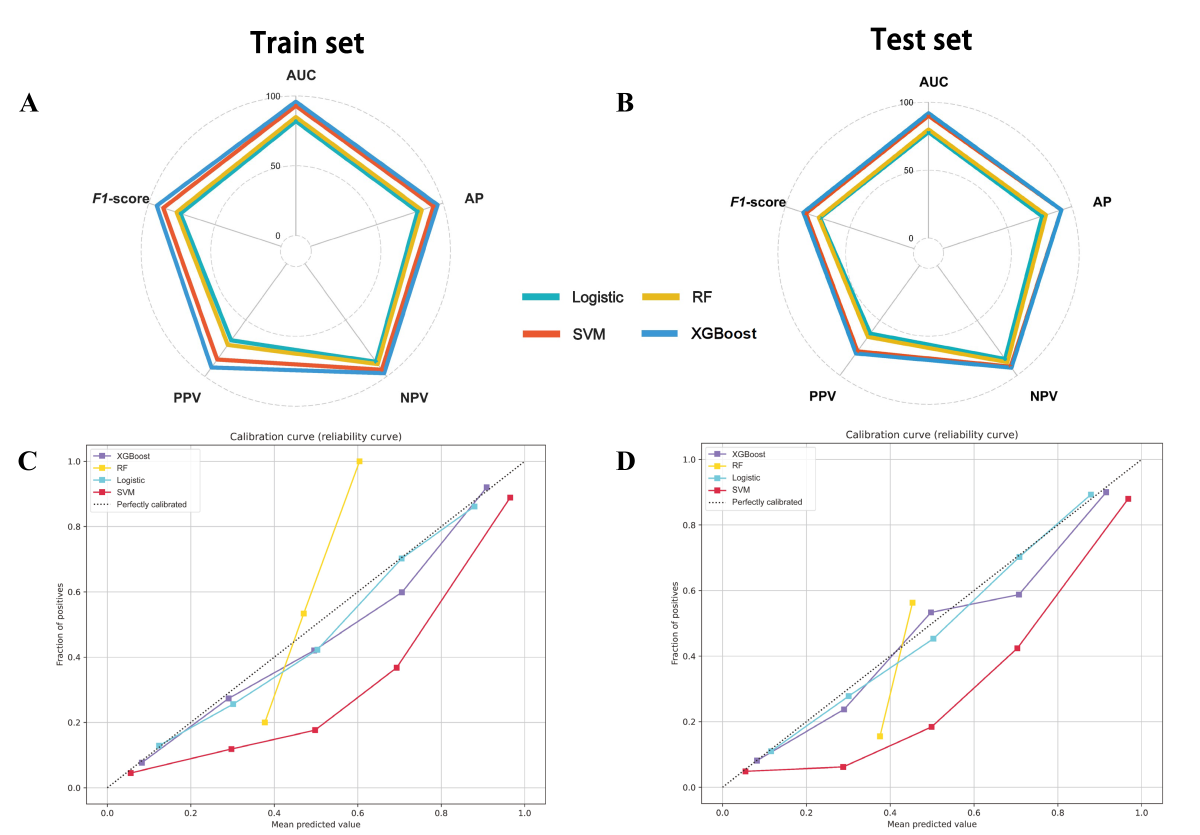
Performance comparison of machine learning models and calibration curves. Calibration curves were used to evaluate the performance of different models. Extreme gradient boosting (XGBoost) demonstrated the best performance in both the train and test sets. (A) Comparison of the performance of multiple machine learning models in the train set, (B) comparison of the performance of multiple machine learning models in the test set, (C) calibration curves of the machine learning models in the train set, and (D) calibration curves of the machine learning models in the test set. AUC: area under the curve; AP: average precision; NPV: negative predictive value; PPV: positive predictive value; RF: random forest; SVM: support vector machine.

Patients were stratified into 3 groups based on the number of CKM risk factors: 0 to 1, 2 to 3, and 4 to 5. Those with more CKM risk factors had a significantly higher risk of MACEs than those in the 0 to 1 risk factor group, indicating that the risk of MACEs increased with the accumulation of CKM risk factors (Figure 3). Multivariate Cox analysis (Table 3) demonstrated that female patients with 4 to 5 CKM risk factors had a 2.49-fold higher risk of MACEs than those with only 0 to 1 risk factor (HR 2.49, 95% CI 1.02-6.09). Similarly, male patients with 4 to 5 CKM risk factors had a 1.48-fold increased risk (HR 1.48, 95% CI 1.07-3.58). In female patients, CKD was the leading attributable risk factor for all-cause mortality, whereas HTG contributed more significantly to mortality in male patients. Furthermore, the interaction between sex and categorized CKM burden (0-1, 2-3, and 4-5) was statistically significant (*P* value for interaction=.03).

**Table 3:** Adjusted hazard ratio (HR) for major adverse cardiovascular events (MACEs) according to the number of joint cardiovascular-kidney-metabolic (CKM) risk factors^a^.

CKM risk factors^b^			MACEs		Participants, n (%)		Adjusted HR (95% CI)		*P* value		*P* for trend	
**Female patients**												.02
	0-1	24		278 (43)		—^c^		—				
	2-3	39		342 (52.1)		1.31 (0.79-2.18)		.30				
	4-5	6		27 (4.2)		2.49 (1.02-6.09)		.04				
**Male patients**												.02
	0-1	90		888 (50.1)		—		—				
	2-3	95		836 (47.2)		1.13 (0.98-1.21)		.25				
	4-5	7		47 (2.7)		1.48 (1.07-3.58)		.03				

^a^All models were adjusted for age, smoking status, history of coronary artery disease, acute coronary syndrome, BMI, low-density lipoprotein cholesterol, number of stents, and SYNTAX score.

^b^For interaction between sex and the number of CKM risk factors, *P*=.03.

^c^Not applicable.

### ML Model Development

**Figure 4:**
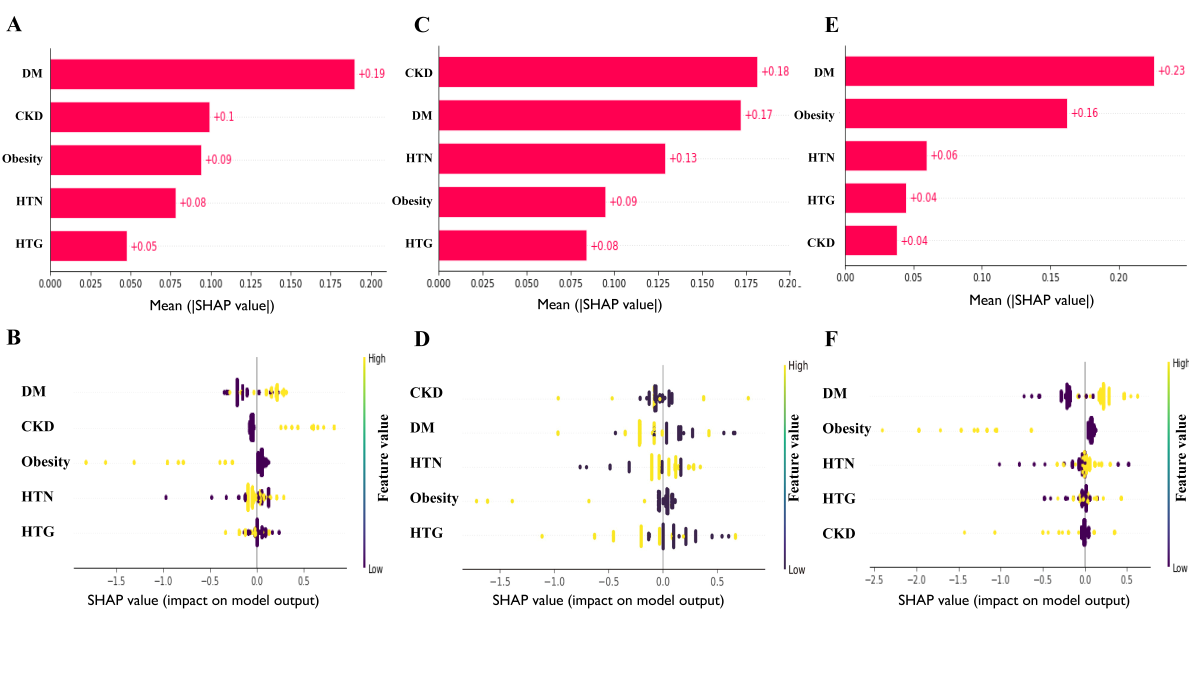
Visualization of the importance of various predictors for major adverse cardiovascular events in patients with moderate-to-severe coronary artery calcification, categorized by sex, using Shapley additive explanations (SHAP) values. (A) Bar plot showing variable importance across the entire cohort, (B) SHAP swarm plot showing variable importance across the entire cohort, (C) bar plot showing the importance of cardiovascular-kidney-metabolic (CKM) risk factors among female participants, (D) SHAP swarm plot showing the importance of CKM risk factors in female participants, (E) bar plot showing the importance of CKM risk factors among male participants, and (F) SHAP swarm plot showing the importance of CKM risk factors in male participants. CKD: chronic kidney disease; DM: diabetes mellitus; HTG: hypertriglyceridemia; HTN: hypertension.

The included patients with MSCAC were randomly divided into a model training cohort and an internal validation cohort in a 7:3 ratio. Four ML models were developed to predict MACEs and compared for their predictive performance. The XGBoost model demonstrated the highest predictive accuracy in the test set, achieving an AUC of 0.92 (95% CI 0.89-0.95), an AP of 0.92 (95% CI 0.89-0.95), an NPV of 0.93 (95% CI 0.90-0.96), a PPV of 0.80 (95% CI 0.75-0.85), and an *F*_1_-score of 0.86 (95% CI 0.82-0.90). Figure 4 illustrates the discriminatory performance of the ML models in the training and test sets. Table S2 in Multimedia Appendix 2 presents a comprehensive array of performance metrics for the various ML models evaluated in this study. Specific details (eg, learning rate and tree depth) are described in Table S3 in Multimedia Appendix 2.

In the external validation cohort, a total of 127 patients with MSCAC were included from January 2021 to June 2021 at a general hospital, of whom 14 (11%) experienced MACEs, and 38 (29.9%) patients were female. Table S4 in Multimedia Appendix 2 presents their baseline characteristics. The XGBoost model demonstrated good performance with an AUC of 0.86 (95% CI 0.78-0.94), an AP of 0.71 (95% CI 0.58-0.84), an NPV of 0.91 (95% CI 0.86-0.96), a PPV of 0.72 (95% CI 0.55-0.89), and an *F*_1_-score of 0.68 (95% CI 0.55-0.81). The calibration curve showed good agreement between predicted and observed probabilities (calibration slope=0.92, 95% CI 0.71-1.13; intercept=0.04, 95% CI –0.12 to 0.20; Brier score=0.09). Moreover, the decision curve analysis indicated its good potential clinical utility (Figure S9 in Multimedia Appendix 1).

### Sex-Specific Importance of CKM Factors

The SHAP algorithm was used to quantify the relative feature importance of each CKM risk factor in the XGBoost model for predicting MACEs. Importantly, SHAP values reflect the contribution of each variable to the model’s predictions and do not imply causality.

In the overall population with MSCAC, baseline DM ranked highest in feature importance for MACEs, with mean SHAP values of 0.23 in male patients and 0.17 in female patients. In female patients, CKD demonstrated the highest feature importance (SHAP value 0.18), followed by DM and hypertension. In contrast, DM remained the most important CKM risk factor in male patients, followed by obesity and hypertension (Figure 4).

Similar sex-specific patterns were observed for other outcomes. For all-cause mortality, CKD ranked highest in feature importance among female patients, whereas HTG showed a greater predictive contribution in male patients. For myocardial infarction, DM was the leading CKM risk factor in female patients, while it ranked second after hypertension in male patients. For unplanned repeat revascularization, DM was the primary CKM risk factor in both sexes; however, HTG exhibited the strongest feature importance in female patients (Figures S10-S12 in Multimedia Appendix 1).

## Discussion

### Principal Findings

This retrospective analysis based on prospective cohort studies highlights significant sex-related differences in the association between CKM risk factors and MACEs among patients with MSCAC undergoing their first PCI. DM demonstrated high feature importance for MACEs in patients of both sexes. Notably, CKD showed a stronger association with MACEs in female patients with MSCAC than in male patients. HTG was more strongly associated with all-cause mortality in male patients with MSCAC, while hypertension demonstrated a more significant association with MACEs in female patients. Additionally, DM was more strongly associated with unplanned repeat revascularization in male patients than in female patients. These sex-specific differences provide valuable insights for identifying high-risk patients with MSCAC and may facilitate more precise, sex-tailored clinical decision-making.

In recent years, there has been growing recognition of the intricate interconnections among cardiovascular, metabolic, and renal diseases, leading to the conceptualization of CKM syndrome [7,8]. CKM is considered a systemic disorder characterized by pathophysiological interactions among metabolic risk factors, CKD, and the cardiovascular system, which collectively contribute to multiorgan dysfunction and a high incidence of adverse cardiovascular outcomes [12]. In this study, we included 5 components: renal function (CKD) and other metabolic risk factors—specifically obesity, hypertension, DM, and HTG—in accordance with the AHA-defined CKM risk factors. A key shared mechanism among these metabolic risk factors is excess and dysfunctional adipose tissue, which leads to inflammation, oxidative stress, and insulin resistance [9,10]. The coexistence of multiple risk factors markedly increases the risk of cardiovascular disease [13,14]. Although lifestyle behaviors, such as smoking, diet, and sleep, are also important determinants of cardiovascular health, they were not included in this study. These behaviors are considered critical for early disease prevention, whereas our focus was on secondary prevention in patients with MSCAC.

CAC has been confirmed to be associated with an increased risk of adverse outcomes in patients with multiple CKM risk factors, including CKD [15] and DM [16]. The accumulation of multiple CKM risk factors accelerates the progression of CAC. Among patients with MSCAC, the coexistence of several metabolic factors further elevates the likelihood of recurrent adverse events. Therefore, it is crucial to emphasize secondary prevention strategies targeting CKM risk factors in this high-risk population.

The distribution of CAC varies by sex, with male patients exhibiting higher levels and prevalence of CAC, and with both calcium content and disease burden increasing steadily with age [17,18]. Similarly, the distribution of CKM syndrome shows marked sex differences. Previous studies indicate that advanced CKM syndrome is more prevalent among adult male patients in the United States (16.9% vs 12.4%) [19] and that in the Chinese population, the weighted prevalence of advanced-stage CKM is higher in male patients than in female patients (24.9% vs 22.3%) [20]. Our study comparing CKM risk factors in male and female patients with MSCAC revealed significant differences: female patients with MSCAC were more likely to possess multiple CKM risk factors (≥4), and the incidence of MACEs was also higher in these female patients. These findings suggest that more intensive management and control of CKM risk factors are particularly important for female patients with MSCAC.

Furthermore, this study explored the differential importance of various CKM risk factors between the sexes. The results indicate that DM ranked highest in feature importance for MACEs in patients with MSCAC, ranking first in the overall population and among male patients, and second among female patients. Previous research has established DM as an independent predictor of increased atherosclerotic burden [21], with patients with DM exhibiting a distinctive pattern of coronary artery atherosclerosis characterized by calcium loads exceeding lipid loads in culprit vessels [22]. Glycemic variability combined with systemic inflammation and oxidative stress has also been shown to be associated with worse clinical outcomes in patients with MSCAC [23]. Interestingly, in female patients with MSCAC, the association between CKD and MACEs was stronger than that observed in male patients, primarily impacting the risk of all-cause mortality. Several factors have been proposed in the literature to explain this observed pattern. For instance, it has been hypothesized that female patients may experience more pronounced reductions in vascular elasticity and calcium salt deposition during vascular calcification, worsening arterial stiffness and increasing the risk of vascular events [24-26]. Second, hormonal changes, such as decreased estrogen levels after menopause, have been suggested to potentially exacerbate calcification and vascular fragility in female patients, further increasing the severity of cardiovascular events and mortality in patients with CKD [27,28]. Additionally, CKD often coexists with metabolic abnormalities and chronic inflammation, which may be more evident or exert more significant effects in female patients, amplifying its impact on all-cause mortality [29,30]. However, these explanations remain speculative and are not directly derived from the SHAP analysis, which only reflects feature importance within the predictive model rather than causal mechanisms. These findings underscore the importance of more targeted management of renal function in female patients with MSCAC. Interventions such as sodium-glucose cotransporter 2 inhibitors or nonsteroidal mineralocorticoid receptor antagonists could potentially help preserve kidney function and improve long-term prognosis in this population [31,32]. Future research should further investigate the biological mechanisms underlying sex disparities to optimize sex-specific risk assessment and intervention strategies.

In the field of cardiovascular research and clinical practice, ML techniques have demonstrated substantial potential [33,34]. Compared with traditional statistical methods, ML algorithms can effectively process large-scale, multidimensional datasets and capture complex nonlinear relationships, thereby improving the accuracy of disease prediction, risk stratification, and personalized treatment strategies [35-38]. Notably, the XGBoost algorithm has shown superior performance in recent cardiovascular-related studies. As an efficient gradient boosting tree method, XGBoost offers advantages in handling missing values, nonlinear features, and resistance to overfitting. Multiple previous studies have validated the superiority of XGBoost in cardiovascular risk prediction—for example, in assessing CAD risk, evaluating heart failure prognosis, and predicting vascular events—achieving better performance than traditional statistical models and other ML techniques [39-42]. In this study, we compared 4 ML models and identified XGBoost as the best-performing model for predicting MACEs based on CKM risk factors. Using SHAP analysis, we quantified the relative feature importance of each CKM risk factor and identified sex-specific patterns of predictive importance. The model was externally validated in an independent cohort, providing preliminary evidence of its generalizability. These findings highlight the utility of ML approaches in elucidating sex differences in the prognostic importance of CKM risk factors and supporting more personalized, sex-tailored clinical decision-making.

This study has several limitations. First, although the ML models were externally validated, this was a single-center retrospective analysis of prospective cohort data conducted exclusively in Chinese patients with MSCAC undergoing PCI. This design may introduce selection bias and limit the generalizability of our findings to other ethnic groups, geographic regions, or health care systems. Second, we evaluated CKM-related indicators only at baseline. Although this approach enabled timely risk assessment, it did not account for potential dynamic changes in these variables over time. Finally, although we adjusted for multiple measured confounders, residual confounding cannot be entirely excluded because of unmeasured or incompletely documented factors, such as detailed medication regimens, menopausal status in female patients, and long-term medication adherence.

### Conclusions

This study systematically evaluated sex differences in the associations between CKM risk factors and adverse prognosis among patients with MSCAC undergoing PCI. Female patients with MSCAC exhibited a higher prevalence of multiple CKM risk factors. DM demonstrated high feature importance for prognosis across both sexes, whereas CKD showed a particularly strong association with adverse outcomes in female patients. By using ML models, particularly XGBoost with SHAP analysis, this study developed effective prognostic prediction tools and identified sex-specific patterns of predictive importance among CKM risk factors. These findings underscore the importance of personalized, sex-tailored management strategies and provide clinicians with valuable insights to optimize care for patients with MSCAC.

## Appendix

Multimedia Appendix 1 Supplementary Figures S1–S12, including patient flowcharts, baseline covariate missing data patterns, cardiovascular-kidney-metabolic risk factor distributions, restricted cubic splines for clinical outcomes, machine learning model validation, and sex-stratified SHAP predictor importance analyses for MSCAC patients.

Multimedia Appendix 2 Supplemental Tables S1-S4 with detailed definitions for various clinical outcomes, comprehensive performance comparisons of several machine learning models, hyperparameter settings for the XGBoost model, and baseline characteristics of coronary artery disease participants within the external validation cohort.

## Abbreviations

AHA: American Heart Association
AP: average precision
AUC: area under the curve
CAC: coronary artery calcification
CAD: coronary artery disease
CKD: chronic kidney disease
CKM: cardiovascular-kidney-metabolic
DM: diabetes mellitus
eGFR: estimated glomerular filtration rate
HbA_1c_: hemoglobin A_1c_
HR: hazard ratio
HTG: hypertriglyceridemia
MACE: major adverse cardiovascular event
ML: machine learning
MSCAC: moderate-to-severe coronary artery calcification
NPV: negative predictive value
PCI: percutaneous coronary intervention
PPV: positive predictive value
SHAP: Shapley additive explanations
SMOTE: synthetic minority oversampling technique
XGBoost: extreme gradient boosting

## Data Availability

The data that support the findings of this study are available from the corresponding author upon reasonable request. The data are not publicly available due to privacy or ethical restrictions.

## References

[1] Onnis C, Virmani R, Kawai K, Nardi V, Lerman A, Cademartiri F, Scicolone R, Boi A, Congiu T, Faa G, Libby P, Saba L (2024). Coronary artery calcification: current concepts and clinical implications. Circulation.

[2] Wang L, Jerosch-Herold M, Jacobs DR Jr, Shahar E, Detrano R, Folsom AR (2006). Coronary artery calcification and myocardial perfusion in asymptomatic adults: the MESA (Multi-Ethnic Study of Atherosclerosis). J Am Coll Cardiol.

[3] Schurgin S, Rich S, Mazzone T (2001). Increased prevalence of significant coronary artery calcification in patients with diabetes. Diabetes Care.

[4] Nakahara T, Dweck MR, Narula N, Pisapia D, Narula J, Strauss HW (2017). Coronary artery calcification: from mechanism to molecular imaging. JACC Cardiovasc Imaging.

[5] Mori H, Torii S, Kutyna M, Sakamoto A, Finn AV, Virmani R (2018). Coronary artery calcification and its progression: what does it really mean?. JACC Cardiovasc Imaging.

[6] Panh L, Lairez O, Ruidavets JB, Galinier M, Carrié D, Ferrières J (2017). Coronary artery calcification: from crystal to plaque rupture. Arch Cardiovasc Dis.

[7] Ndumele CE, Rangaswami J, Chow SL, Neeland IJ, Tuttle KR, Khan SS, Coresh J, Mathew RO, Baker-Smith CM, Carnethon MR, Despres JP, Ho JE, Joseph JJ, Kernan WN, Khera A, Kosiborod MN, Lekavich CL, Lewis EF, Lo KB, Ozkan B, Palaniappan LP, Patel SS, Pencina MJ, Powell-Wiley TM, Sperling LS, Virani SS, Wright JT, Rajgopal Singh R, Elkind MS (2023). Cardiovascular-kidney-metabolic health: a presidential advisory from the American Heart Association. Circulation.

[8] Ndumele CE, Neeland IJ, Tuttle KR, Chow SL, Mathew RO, Khan SS, Coresh J, Baker-Smith CM, Carnethon MR, Després JP, Ho JE, Joseph JJ, Kernan WN, Khera A, Kosiborod MN, Lekavich CL, Lewis EF, Lo KB, Ozkan B, Palaniappan LP, Patel SS, Pencina MJ, Powell-Wiley TM, Sperling LS, Virani SS, Wright JT, Rajgopal Singh R, Elkind MS, Rangaswami J (2023). A synopsis of the evidence for the science and clinical management of cardiovascular-kidney-metabolic (CKM) syndrome: a scientific statement from the American Heart Association. Circulation.

[9] Eckel RH, Grundy SM, Zimmet PZ (2005). The metabolic syndrome. Lancet.

[10] Cornier MA, Dabelea D, Hernandez TL, Lindstrom RC, Steig AJ, Stob NR, Van Pelt RE, Wang H, Eckel RH (2008). The metabolic syndrome. Endocr Rev.

[11] Levey AS, Stevens LA, Schmid CH, Zhang YL, Castro AF 3rd, Feldman HI, Kusek JW, Eggers P, Van Lente F, Greene T, Coresh J (2009). A new equation to estimate glomerular filtration rate. Ann Intern Med.

[12] Quaggin SE, Magod B (2024). A united vision for cardiovascular-kidney-metabolic health. Nat Rev Nephrol.

[13] Mottillo S, Filion KB, Genest J, Joseph L, Pilote L, Poirier P, Rinfret S, Schiffrin EL, Eisenberg MJ (2010). The metabolic syndrome and cardiovascular risk a systematic review and meta-analysis. J Am Coll Cardiol.

[14] Li J, Wei X (2025). Association of cardiovascular-kidney-metabolic syndrome with all-cause and cardiovascular mortality: a prospective cohort study. Am J Prev Cardiol.

[15] Jung CY, Yun HR, Park JT, Joo YS, Kim HW, Yoo TH, Kang SW, Lee J, Chae DW, Chung W, Kim YS, Oh KH, Han SH (2023). Association of coronary artery calcium with adverse cardiovascular outcomes and death in patients with chronic kidney disease: results from the KNOW-CKD. Nephrol Dial Transplant.

[16] Malik S, Zhao Y, Budoff M, Nasir K, Blumenthal RS, Bertoni AG, Wong ND (2017). Coronary artery calcium score for long-term risk classification in individuals with type 2 diabetes and metabolic syndrome from the multi-ethnic study of atherosclerosis. JAMA Cardiol.

[17] McClelland RL, Chung H, Detrano R, Post W, Kronmal RA (2006). Distribution of coronary artery calcium by race, gender, and age: results from the Multi-Ethnic Study of Atherosclerosis (MESA). Circulation.

[18] Javaid A, Dardari ZA, Mitchell JD, Whelton SP, Dzaye O, Lima JA, Lloyd-Jones DM, Budoff M, Nasir K, Berman DS, Rumberger J, Miedema MD, Villines TC, Blaha MJ (2022). Distribution of coronary artery calcium by age, sex, and race among patients 30-45 years old. J Am Coll Cardiol.

[19] Aggarwal R, Ostrominski JW, Vaduganathan M (2024). Prevalence of cardiovascular-kidney-metabolic syndrome stages in US adults, 2011-2020. JAMA.

[20] Zheng C, Cai A, Sun M, Wang X, Song Q, Pei X, Cao X, Tian Y, Lip GY, Parati G, Wang Z, Feng Y, Zhou Z (2025). Prevalence and mortality of cardiovascular-kidney-metabolic syndrome in China: a nationwide population-based study. JACC Asia.

[21] Mintz GS, Painter JA, Pichard AD, Kent KM, Satler LF, Popma JJ, Chuang YC, Bucher TA, Sokolowicz LE, Leon MB (1995). Atherosclerosis in angiographically "normal" coronary artery reference segments: an intravascular ultrasound study with clinical correlations. J Am Coll Cardiol.

[22] Niccoli G, Giubilato S, Di Vito L, Leo A, Cosentino N, Pitocco D, Marco V, Ghirlanda G, Prati F, Crea F (2013). Severity of coronary atherosclerosis in patients with a first acute coronary event: a diabetes paradox. Eur Heart J.

[23] Lin Z, Song Y, Yuan S, He J, Dou K (2024). Prognostic value of the stress-hyperglycaemia ratio in patients with moderate-to-severe coronary artery calcification: insights from a large cohort study. Diabetes Obes Metab.

[24] Ren SC, Mao N, Yi S, Ma X, Zou JQ, Tang X, Fan JM (2022). Vascular calcification in chronic kidney disease: an update and perspective. Aging Dis.

[25] Ward LJ, Laucyte-Cibulskiene A, Hernandez L, Ripsweden J, Stenvinkel P, Kublickiene K, GOING-FWD Collaborators (2023). Coronary artery calcification and aortic valve calcification in patients with kidney failure: a sex-disaggregated study. Biol Sex Differ.

[26] GBD Chronic Kidney Disease Collaboration (2020). Global, regional, and national burden of chronic kidney disease, 1990-2017: a systematic analysis for the Global Burden of Disease Study 2017. Lancet.

[27] Suzuki H, Kondo K (2012). Chronic kidney disease in postmenopausal women. Hypertens Res.

[28] West SL, Swan VJ, Jamal SA (2010). Effects of calcium on cardiovascular events in patients with kidney disease and in a healthy population. Clin J Am Soc Nephrol.

[29] Laucyte-Cibulskiene A, Ward LJ, Ebert T, Tosti G, Tucci C, Hernandez L, Kautzky-Willer A, Herrero MT, Norris CM, Pilote L, Söderberg M, Brismar TB, Ripsweden J, Stenvinkel P, Raparelli V, Kublickiene K (2021). Role of GDF-15, YKL-40 and MMP 9 in patients with end-stage kidney disease: focus on sex-specific associations with vascular outcomes and all-cause mortality. Biol Sex Differ.

[30] Wang XR, Zhang JJ, Xu XX, Wu YG (2019). Prevalence of coronary artery calcification and its association with mortality, cardiovascular events in patients with chronic kidney disease: a systematic review and meta-analysis. Ren Fail.

[31] de Boer IH, Khunti K, Sadusky T, Tuttle KR, Neumiller JJ, Rhee CM, Rosas SE, Rossing P, Bakris G (2022). Diabetes management in chronic kidney disease: a consensus report by the American Diabetes Association (ADA) and Kidney Disease: Improving Global Outcomes (KDIGO). Diabetes Care.

[32] Agarwal R, Filippatos G, Pitt B, Anker SD, Rossing P, Joseph A, Kolkhof P, Nowack C, Gebel M, Ruilope LM, Bakris GL (2022). Cardiovascular and kidney outcomes with finerenone in patients with type 2 diabetes and chronic kidney disease: the FIDELITY pooled analysis. Eur Heart J.

[33] Deo RC (2024). Artificial intelligence and machine learning in cardiology. Circulation.

[34] D'Ascenzo F, De Filippo O, Gallone G, Mittone G, Deriu MA, Iannaccone M, Ariza-Solé A, Liebetrau C, Manzano-Fernández S, Quadri G, Kinnaird T, Campo G, Simao Henriques JP, Hughes JM, Dominguez-Rodriguez A, Aldinucci M, Morbiducci U, Patti G, Raposeiras-Roubin S, Abu-Assi E, De Ferrari GM (2021). Machine learning-based prediction of adverse events following an acute coronary syndrome (PRAISE): a modelling study of pooled datasets. Lancet.

[35] Al'Aref SJ, Anchouche K, Singh G, Slomka PJ, Kolli KK, Kumar A, Pandey M, Maliakal G, van Rosendael AR, Beecy AN, Berman DS, Leipsic J, Nieman K, Andreini D, Pontone G, Schoepf UJ, Shaw LJ, Chang HJ, Narula J, Bax JJ, Guan Y, Min JK (2019). Clinical applications of machine learning in cardiovascular disease and its relevance to cardiac imaging. Eur Heart J.

[36] Augusto JB, Davies RH, Bhuva AN, Knott KD, Seraphim A, Alfarih M, Lau C, Hughes RK, Lopes LR, Shiwani H, Treibel TA, Gerber BL, Hamilton-Craig C, Ntusi NA, Pontone G, Desai MY, Greenwood JP, Swoboda PP, Captur G, Cavalcante J, Bucciarelli-Ducci C, Petersen SE, Schelbert E, Manisty C, Moon JC (2021). Diagnosis and risk stratification in hypertrophic cardiomyopathy using machine learning wall thickness measurement: a comparison with human test-retest performance. Lancet Digit Health.

[37] Ouyang D, Cheng S (2022). Revival and revision of right ventricular assessment by machine learning. JACC Cardiovasc Imaging.

[38] Ng AC, Delgado V, Bax JJ (2020). Individualized patient risk stratification using machine learning and topological data analysis. JACC Cardiovasc Imaging.

[39] Unterhuber M, Kresoja KP, Rommel K, Besler C, Baragetti A, Klöting N, Ceglarek U, Blüher M, Scholz M, Catapano AL, Thiele H, Lurz P (2021). Proteomics-enabled deep learning machine algorithms can enhance prediction of mortality. J Am Coll Cardiol.

[40] Ouwerkerk W, Belo Pereira JP, Maasland T, Emmens JE, Figarska SM, Tromp J, Koekemoer AL, Nelson CP, Nath M, Romaine SP, Cleland JG, Zannad F, van Veldhuisen DJ, Lang CC, Ponikowski P, Filippatos G, Anker S, Metra M, Dickstein K, Ng LL, de Boer RA, van Riel N, Nieuwdorp M, Groen AK, Stroes E, Zwinderman AH, Samani NJ, Lam CS, Levin E, Voors AA (2023). Multiomics analysis provides novel pathways related to progression of heart failure. J Am Coll Cardiol.

[41] Kalmady SV, Salimi A, Sun W, Sepehrvand N, Nademi Y, Bainey K, Ezekowitz J, Hindle A, McAlister F, Greiner R, Sandhu R, Kaul P (2024). Development and validation of machine learning algorithms based on electrocardiograms for cardiovascular diagnoses at the population level. NPJ Digit Med.

[42] Wang Z, Gu Y, Huang L, Liu S, Chen Q, Yang Y, Hong G, Ning W (2024). Construction of machine learning diagnostic models for cardiovascular pan-disease based on blood routine and biochemical detection data. Cardiovasc Diabetol.

