# Supplementary material for: Machine Learning Analysis of Sex Differences in Cardiovascular-Kidney-Metabolic Risk Factors and Prognosis Among Patients With Moderate-to-Severe Coronary Artery Calcification: Prospective Cohort Study
Source: J Med Internet Res. 2026 Jul 16;28:e82742. doi: 10.2196/82742 (PMC13424754; doi:10.2196/82742)
Supplement: Multimedia Appendix 1 [file jmir_v28i1e82742_app1.pdf]

Supplementary Figure

Figure S1 Flowchart of MSCAC patients included. MSCAC, moderate to severe coronary artery calcification; PCI, percutaneous coronary intervention.

Figure S2 Flowchart of MSCAC patients included in the external validation set. MSCAC, moderate to severe coronary artery calcification; PCI, percutaneous coronary intervention.

Figure S3 Missing data patterns in baseline covariates visually.

Figure S4 Venn diagram demonstrating the distribution of different CKM risk factors in the overall population. CKD, chronic kidney disease; HTN, hypertension; DM, diabetes mellitus; HTG, hypertriglyceridemia.

Figure S5 Restricted cubic spline analysis illustrating the nonlinear or linear relationships between various CKM risk factors— including eGFR, BMI, HbA1c, SBP, TG— and MACE. MACE, major adverse cardiovascular events; HR, hazard ratio; CI, confidence interval; eGFR, estimated glomerular filtration rate; BMI, body mass index; SBP, systolic blood pressure; TG, triglyceride.

Figure S6 Restricted cubic spline analysis demonstrating the nonlinear or linear associations between CKM risk factors (eGFR, BMI, HbA1c, SBP, TG) and all-cause mortality. HR, hazard ratio; CI, confidence interval; eGFR, estimated glomerular filtration rate; BMI, body mass index; SBP, systolic blood pressure; TG, triglyceride.

Figure S7 Restricted cubic spline analysis showing the relationships between CKM risk factors (eGFR, BMI, HbA1c, SBP, TG) and non-fatal myocardial infarction. MI, myocardial infarction, HR, hazard ratio; CI, confidence interval; eGFR, estimated glomerular filtration rate; BMI, body mass index; SBP, systolic blood pressure; TG, triglyceride.

Figure S8 Restricted cubic spline analysis depicting the associations between CKM risk factors (eGFR, BMI, HbA1c, SBP, TG) and unplanned repeat revascularization. HR, hazard ratio; CI, confidence interval; eGFR, estimated glomerular filtration rate; BMI, body mass index; SBP, systolic blood pressure; TG, triglyceride.

Figure S9 Performance of machine learning model in the external validation set. (A) ROC curve and (B) precision and recall curve of XGB. (C) Calibration curve indicated a certain level of reliability of XGB. (D) DCA curve indicated its good potential clinical utility. XGB, EXtreme Gradient Boosting; DCA, decision curve analysis.

Figure S10 Visualization of predictor importance for all-cause mortality in patients with MSCAC by SHAP, categorized by sex. Panels A and B display variable importance in the entire cohort via bar and swarm plots, respectively. Panels C and D show CKM risk factor importance in females, while Panels E and F present the same for males. CKM, Cardiovascular-Kidney-Metabolic; CKD, chronic kidney disease; HTN, hypertension; DM, diabetes mellitus; HTG, hypertriglyceridemia; MSCAC, moderate to severe coronary artery calcification.

Figure S11 Visualization of predictor importance for myocardial infarction in patients with MSCAC by SHAP, categorized by sex. Variants include bar and swarm plots for the whole cohort (A, B), females (C, D), and males (E, F). CKM, Cardiovascular-Kidney-Metabolic; CKD, chronic kidney disease; HTN, hypertension; DM, diabetes mellitus; HTG, hypertriglyceridemia; MI, myocardial infarction; MSCAC, moderate to severe coronary artery calcification.

Figure S12 Visualization of predictor importance for unplanned repeat revascularization in patients with MSCAC by SHAP, categorized by sex. The plots follow the same format as above, with panels A–F representing the entire cohort, females, and males respectively. CKM, Cardiovascular-Kidney-Metabolic; CKD, chronic kidney disease; HTN, hypertension; DM, diabetes mellitus; HTG, hypertriglyceridemia; MSCAC, moderate to severe coronary artery calcification.

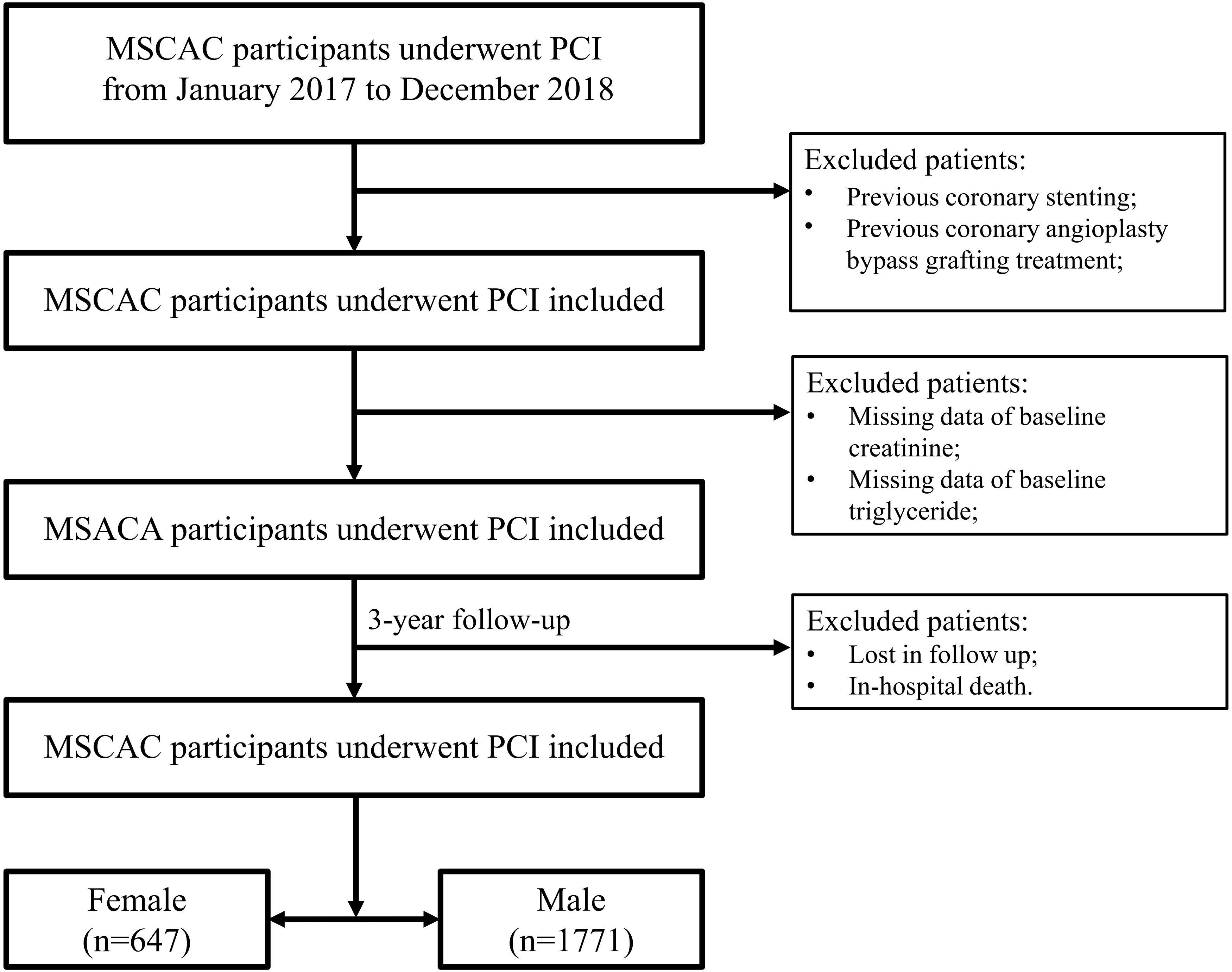
MSCAC participants underwent PCI from January 2017 to December 2018
Excluded patients:
• Previous coronary stenting;
• Previous coronary angioplasty bypass grafting treatment;
MSCAC participants underwent PCI included
Excluded patients:
• Missing data of baseline creatinine;
• Missing data of baseline triglyceride;
MSACA participants underwent PCI included
3-year follow-up
Excluded patients:
• Lost in follow up;
• In-hospital death.
MSCAC participants underwent PCI included
Female (n=647)
Male (n=1771)

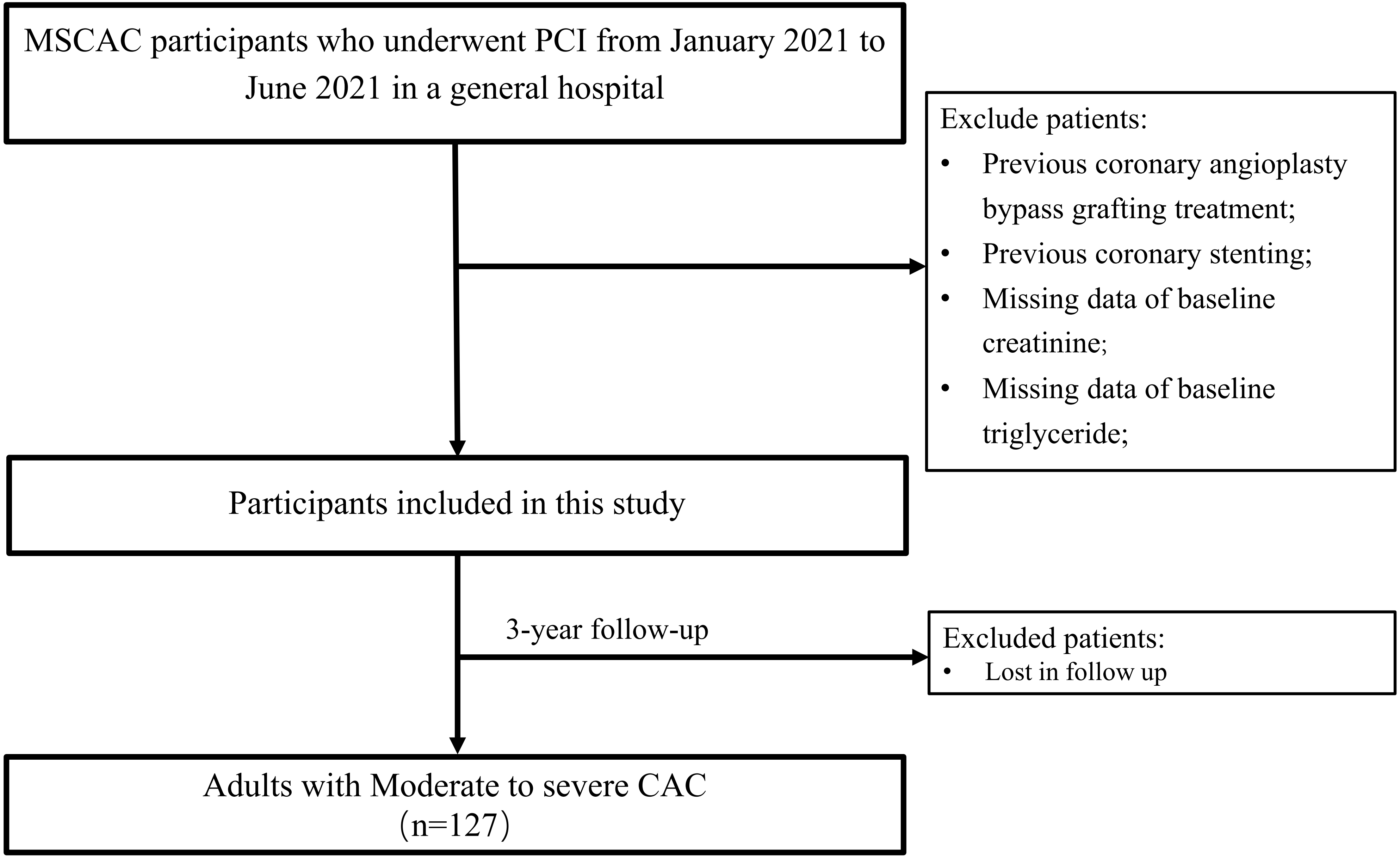
MSCAC participants who underwent PCI from January 2021 to June 2021 in a general hospital
Exclude patients:
• Previous coronary angioplasty bypass grafting treatment;
• Previous coronary stenting;
• Missing data of baseline creatinine;
• Missing data of baseline triglyceride;
Participants included in this study
3-year follow-up
Excluded patients:
• Lost in follow up
Adults with Moderate to severe CAC (n=127)

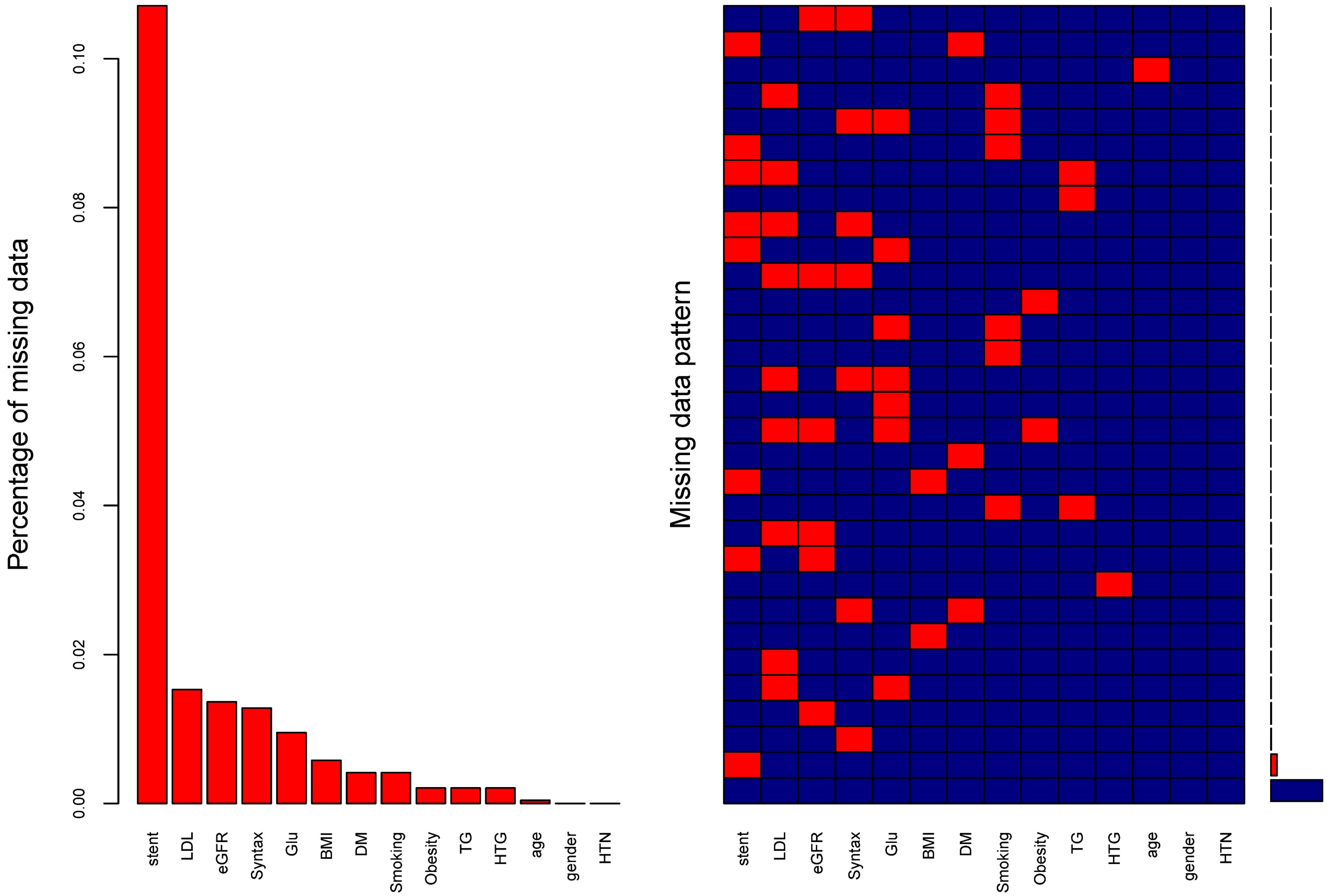

Percentage of missing data
0.00
0.02
0.04
0.06
0.08
0.10
stent
LDL
eGFR
Syntax
Glu
BMI
DM
Smoking
Obesity
TG
HTG
age
gender
HTN
Missing data pattern
stent
LDL
eGFR
Syntax
Glu
BMI
DM
Smoking
Obesity
TG
HTG
age
gender
HTN

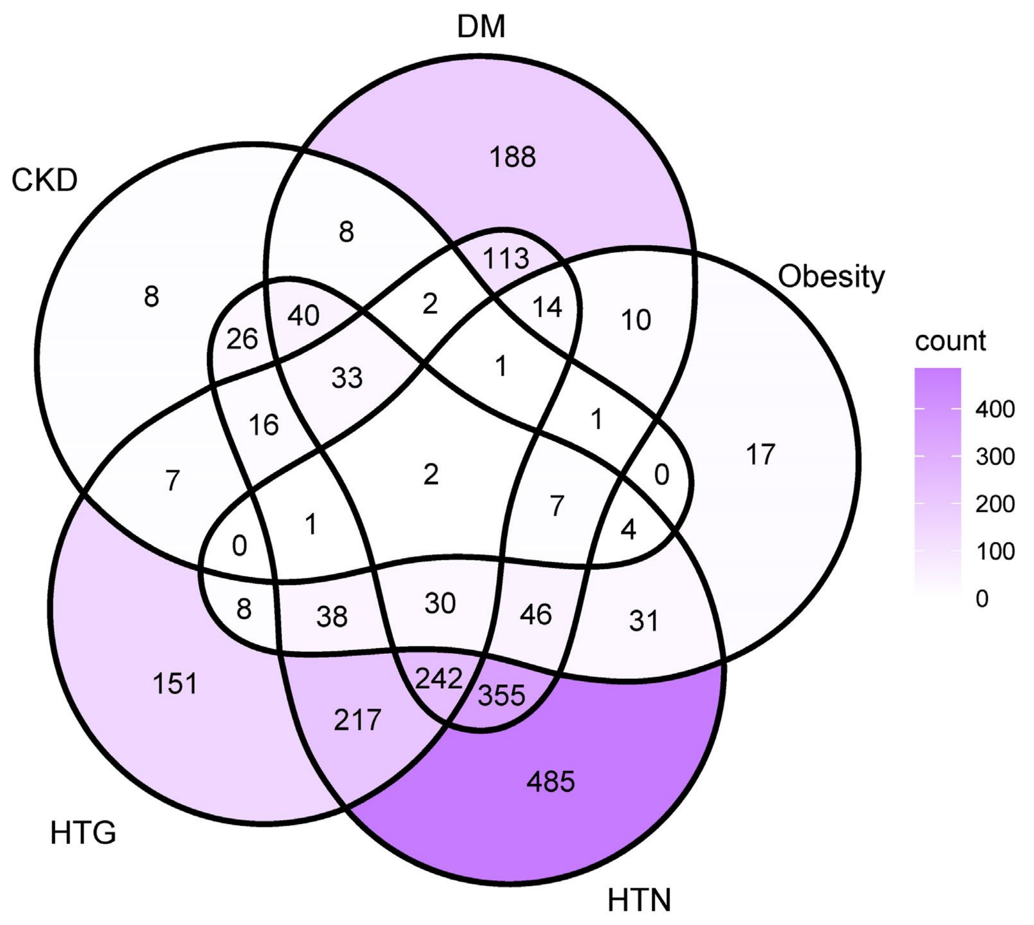

DM
CKD
Obesity
HTG
HTN
count
400
300
200
100
0
188
8
8
113
2
14
10
26
40
33
1
16
1
7
2
0
17
0
1
7
4
8
38
30
46
31
151
242
355
217
485

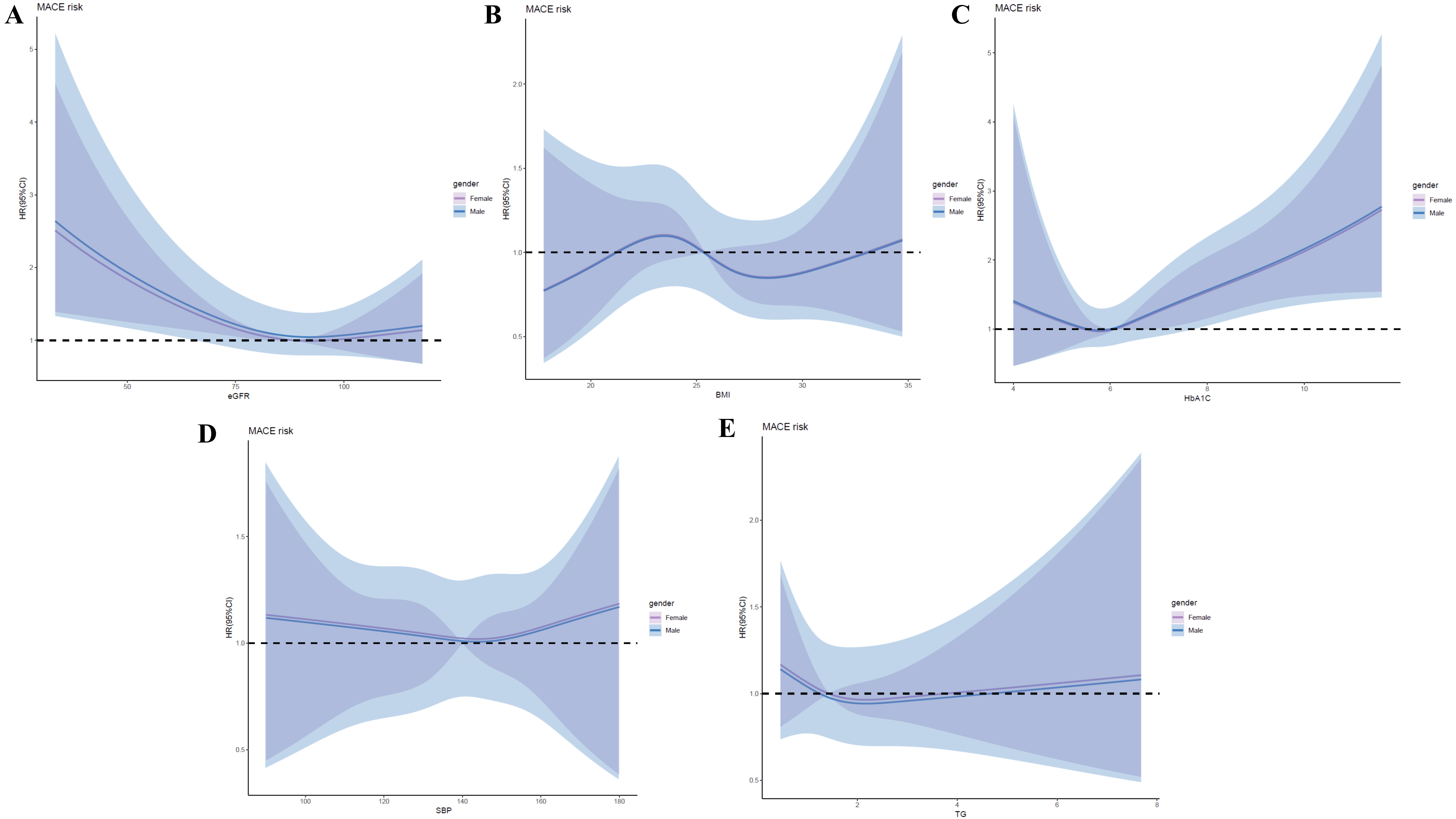

A
MACE risk
HR(95%CI)
eGFR
gender
Female
Male
B
MACE risk
HR(95%CI)
BMI
gender
Female
Male
C
MACE risk
HR(95%CI)
HbA1C
gender
Female
Male
D
MACE risk
HR(95%CI)
SBP
gender
Female
Male
E
MACE risk
HR(95%CI)
TG
gender
Female
Male

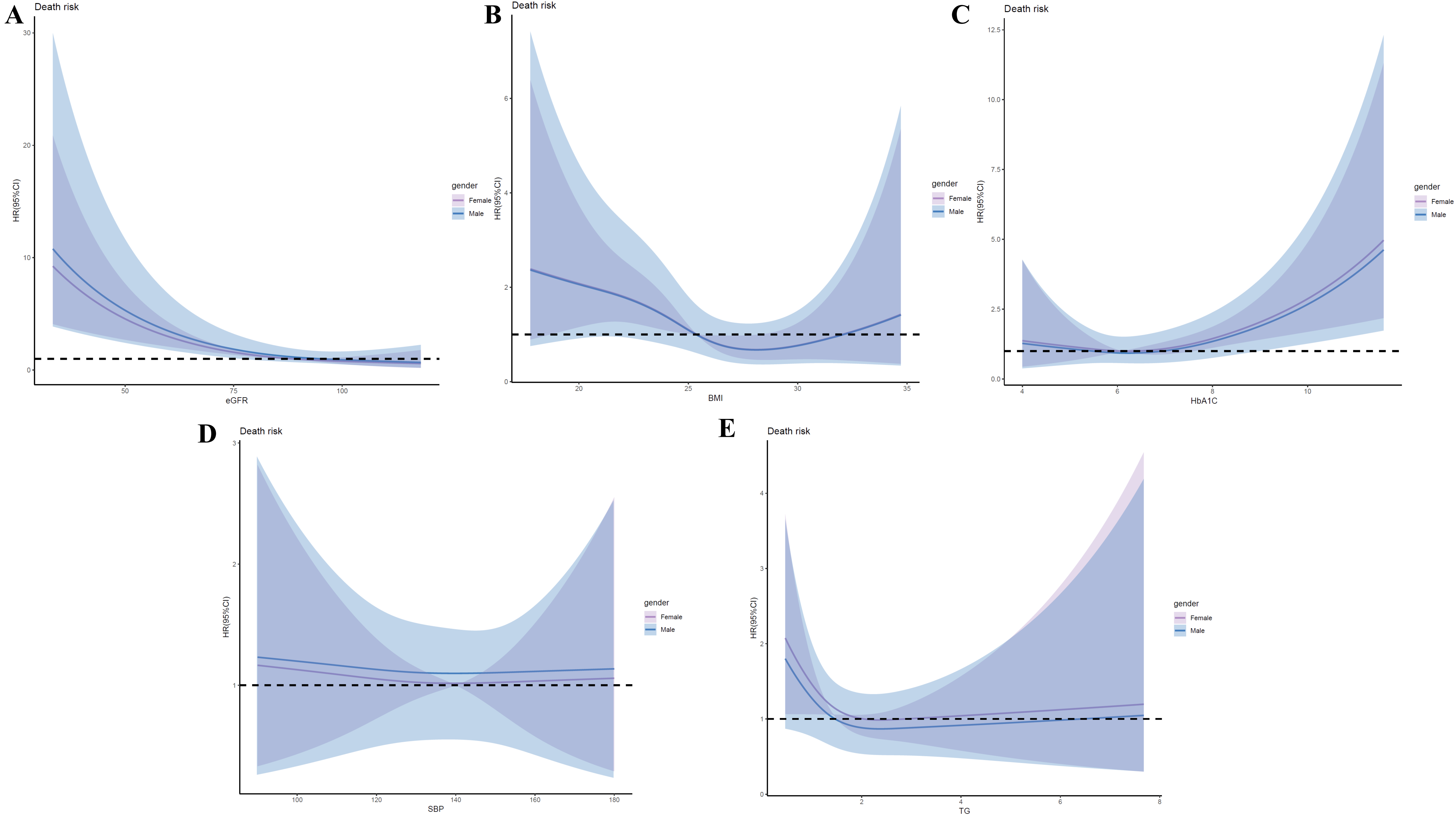
A
Death risk
HR(95%CI)
30
20
10
0
50
75
100
eGFR
gender
Female
Male
B
Death risk
HR(95%CI)
6
4
2
0
20
25
30
35
BMI
gender
Female
Male
C
Death risk
HR(95%CI)
12.5
10.0
7.5
5.0
2.5
0.0
4
6
8
10
HbA1C
gender
Female
Male
D
Death risk
HR(95%CI)
3
2
1
100
120
140
160
180
SBP
gender
Female
Male
E
Death risk
HR(95%CI)
4
3
2
1
0
2
4
6
8
TG
gender
Female
Male

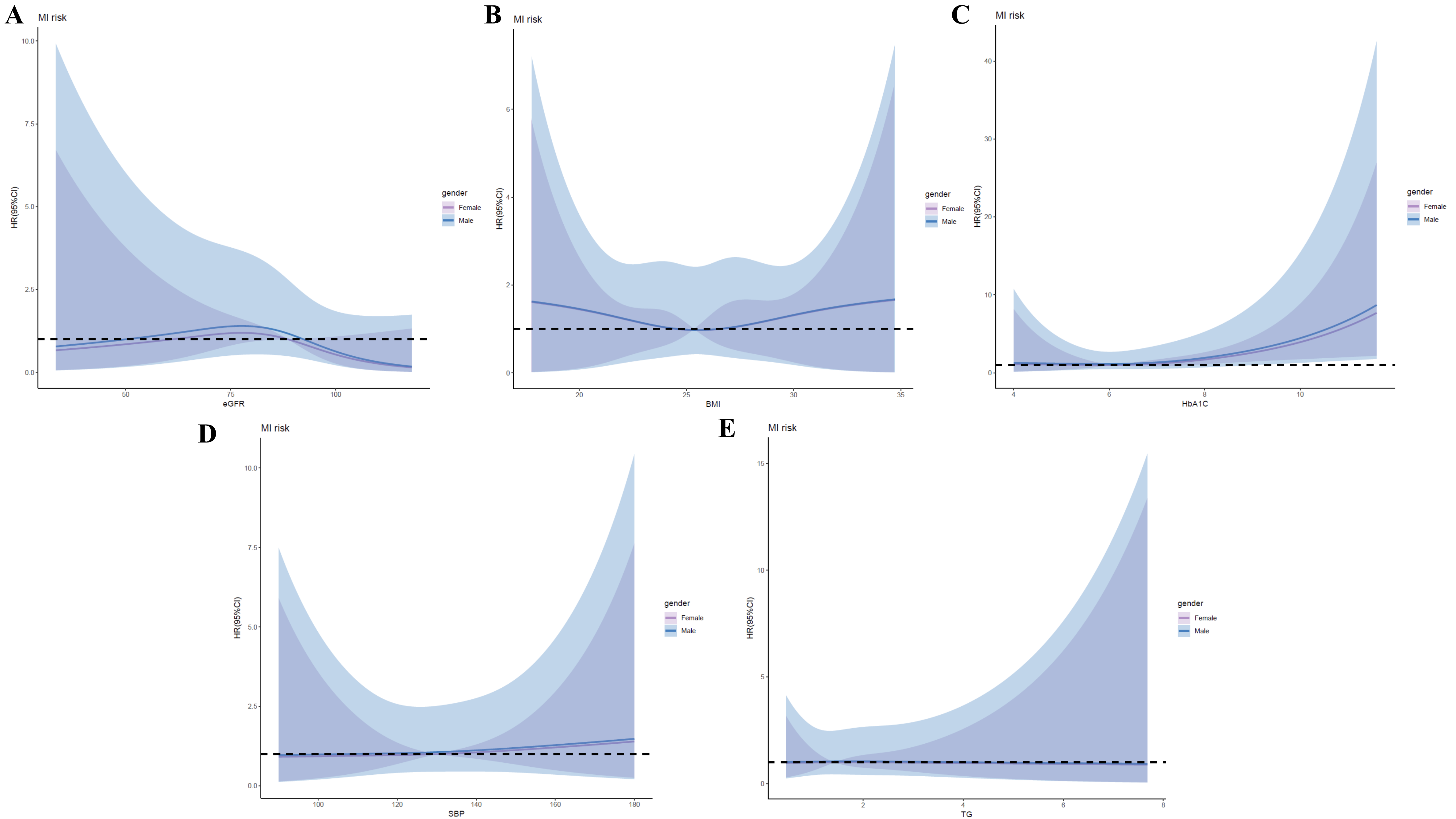

A
MI risk
HR(95%CI)
eGFR
B
MI risk
HR(95%CI)
BMI
C
MI risk
HR(95%CI)
HbA1C
D
MI risk
HR(95%CI)
SBP
E
MI risk
HR(95%CI)
TG
gender
Female
Male

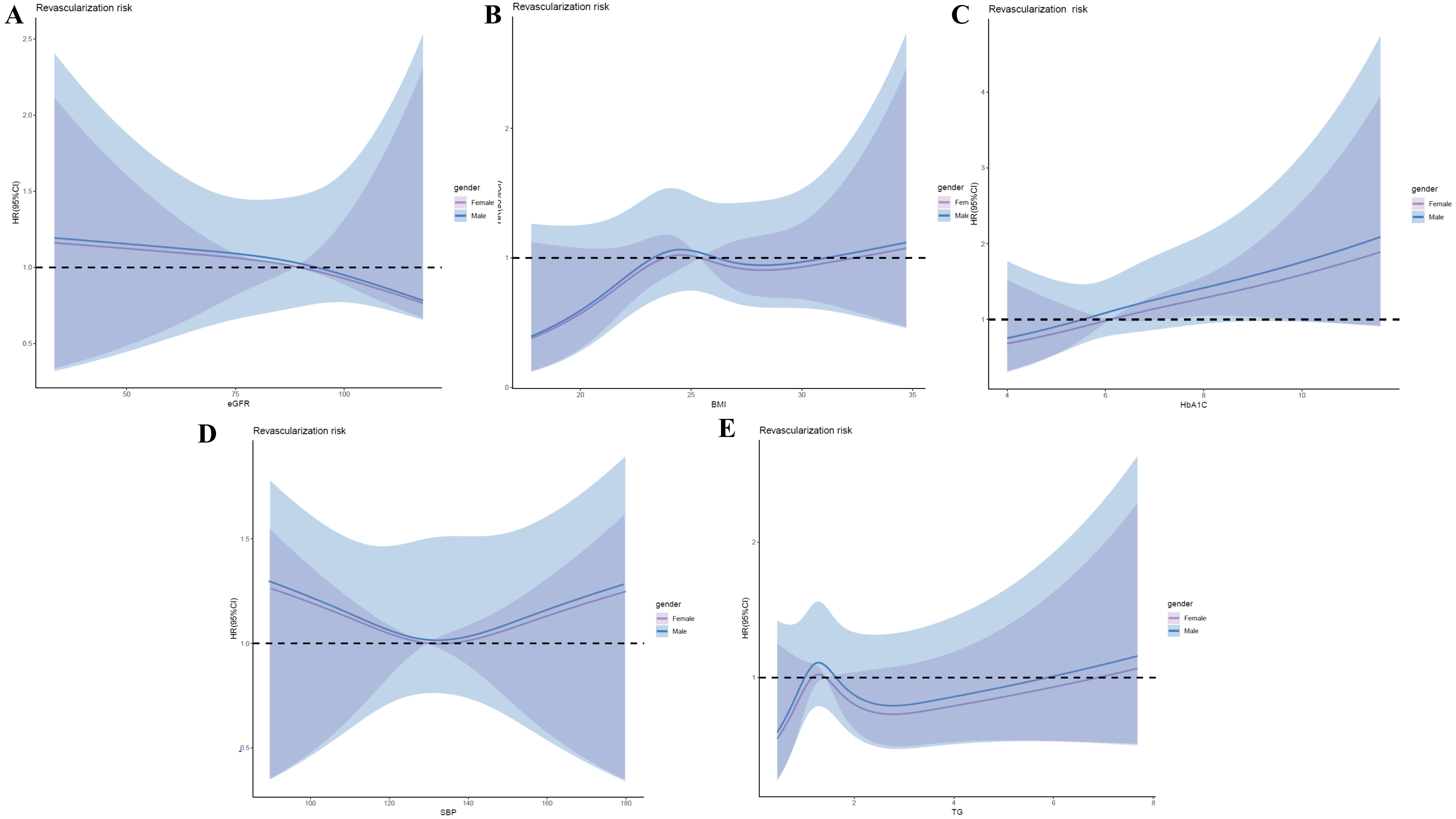

A
Revascularization risk
HR(95%CI)
eGFR
gender
Female
Male
B
Revascularization risk
BMI
gender
Female
Male
C
Revascularization risk
HR(95%CI)
HbA1C
gender
Female
Male
D
Revascularization risk
HR(95%CI)
SBP
gender
Female
Male
E
Revascularization risk
HR(95%CI)
TG
gender
Female
Male

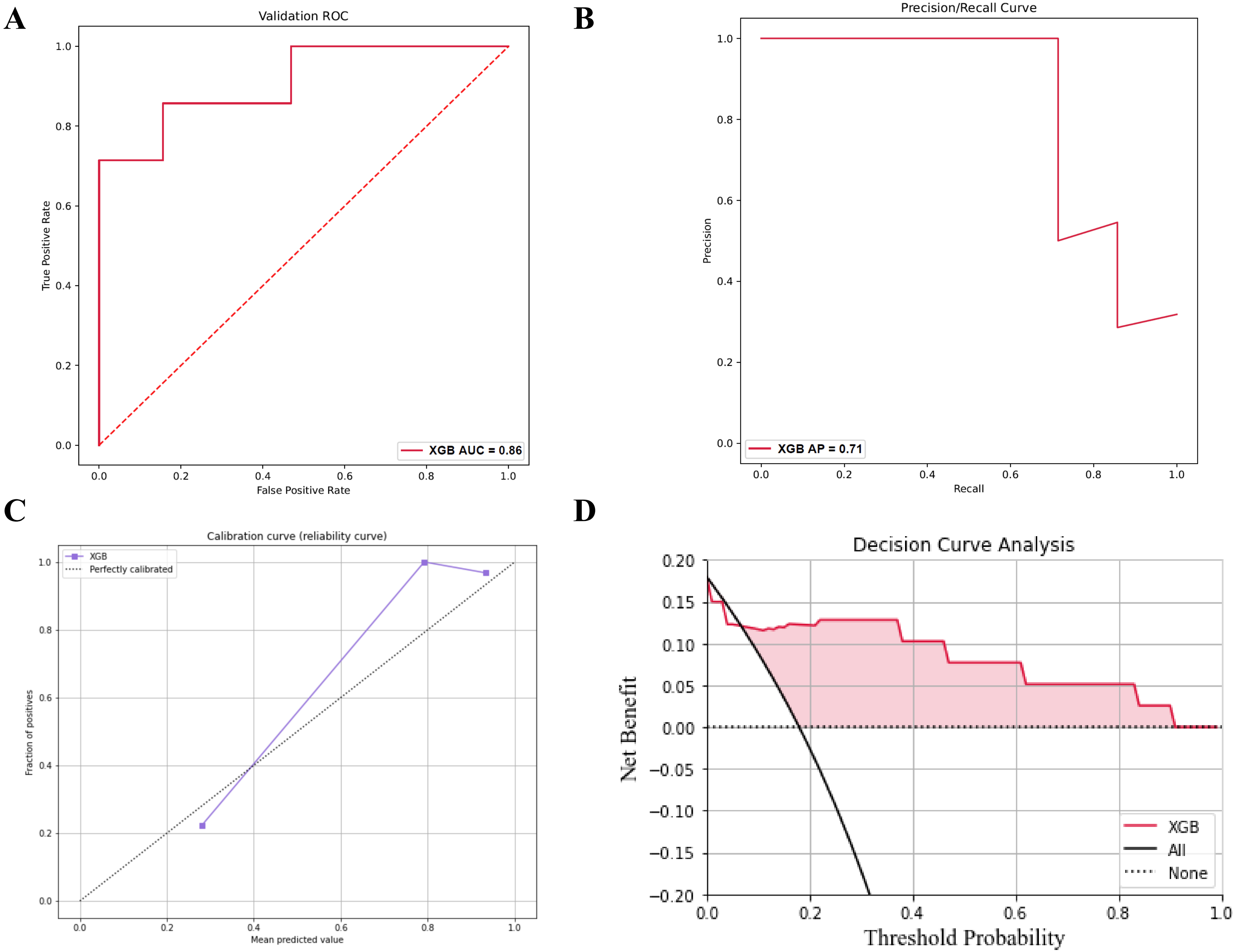
A
Validation ROC
True Positive Rate
False Positive Rate
XGB AUC = 0.86
0.0
0.2
0.4
0.6
0.8
1.0
B
Precision/Recall Curve
Precision
Recall
XGB AP = 0.71
C
Calibration curve (reliability curve)
Fraction of positives
Mean predicted value
XGB
Perfectly calibrated
D
Decision Curve Analysis
Net Benefit
Threshold Probability
0.20
0.15
0.10
0.05
0.00
−0.05
−0.10
−0.15
−0.20
XGB
All
None

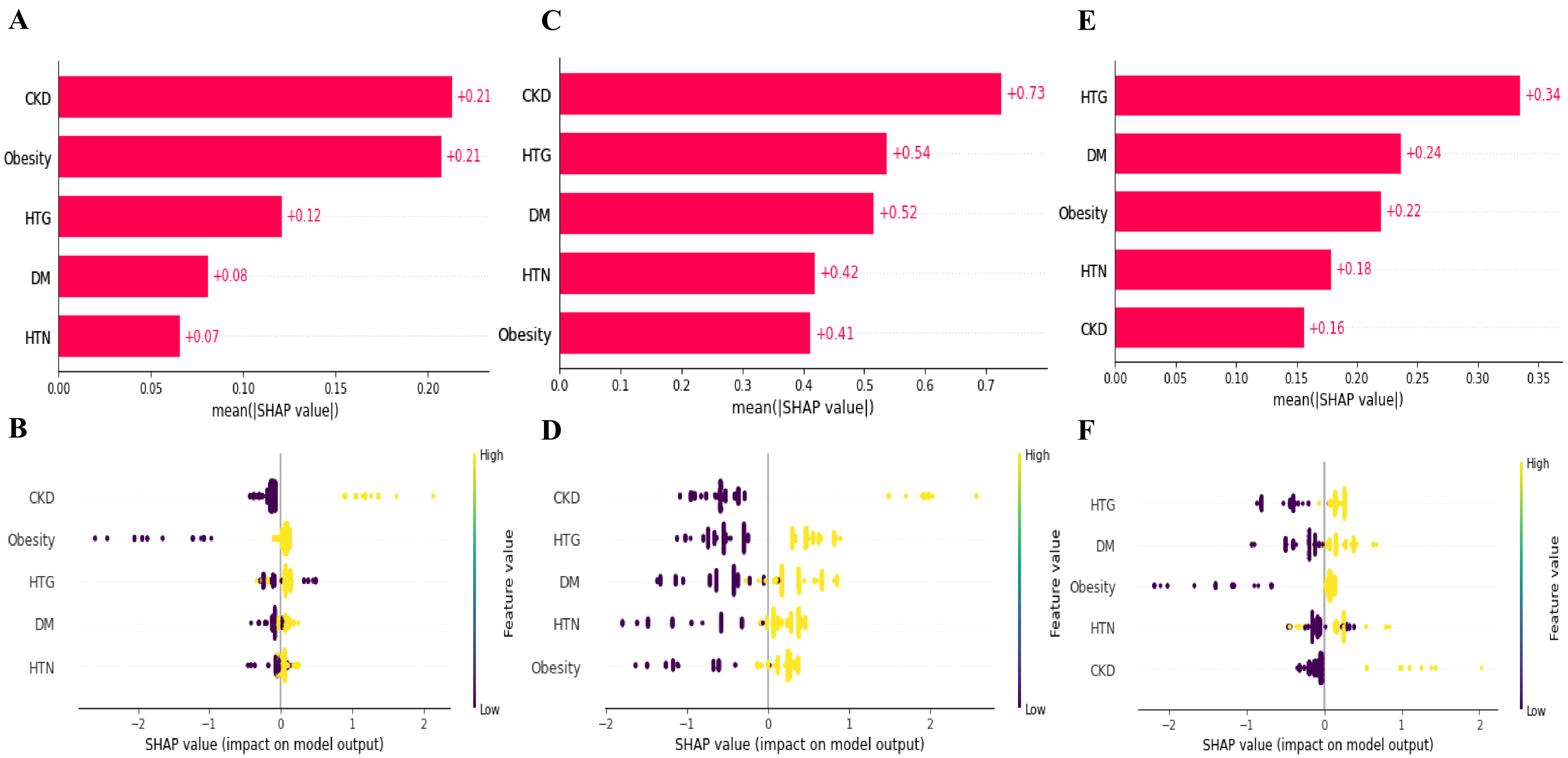

A
CKD
Obesity
HTG
DM
HTN
+0.21
+0.21
+0.12
+0.08
+0.07
0.00
0.05
0.10
0.15
0.20
mean(|SHAP value|)
C
CKD
HTG
DM
HTN
Obesity
+0.73
+0.54
+0.52
+0.42
+0.41
0.0
0.1
0.2
0.3
0.4
0.5
0.6
0.7
mean(|SHAP value|)
E
HTG
DM
Obesity
HTN
CKD
+0.34
+0.24
+0.22
+0.18
+0.16
0.00
0.05
0.10
0.15
0.20
0.25
0.30
0.35
mean(|SHAP value|)
B
CKD
Obesity
HTG
DM
HTN
−2
−1
0
1
2
SHAP value (impact on model output)
High
Low
Feature value
D
CKD
HTG
DM
HTN
Obesity
−2
−1
0
1
2
SHAP value (impact on model output)
High
Low
Feature value
F
HTG
DM
Obesity
HTN
CKD
−2
−1
0
1
2
SHAP value (impact on model output)
High
Low
Feature value

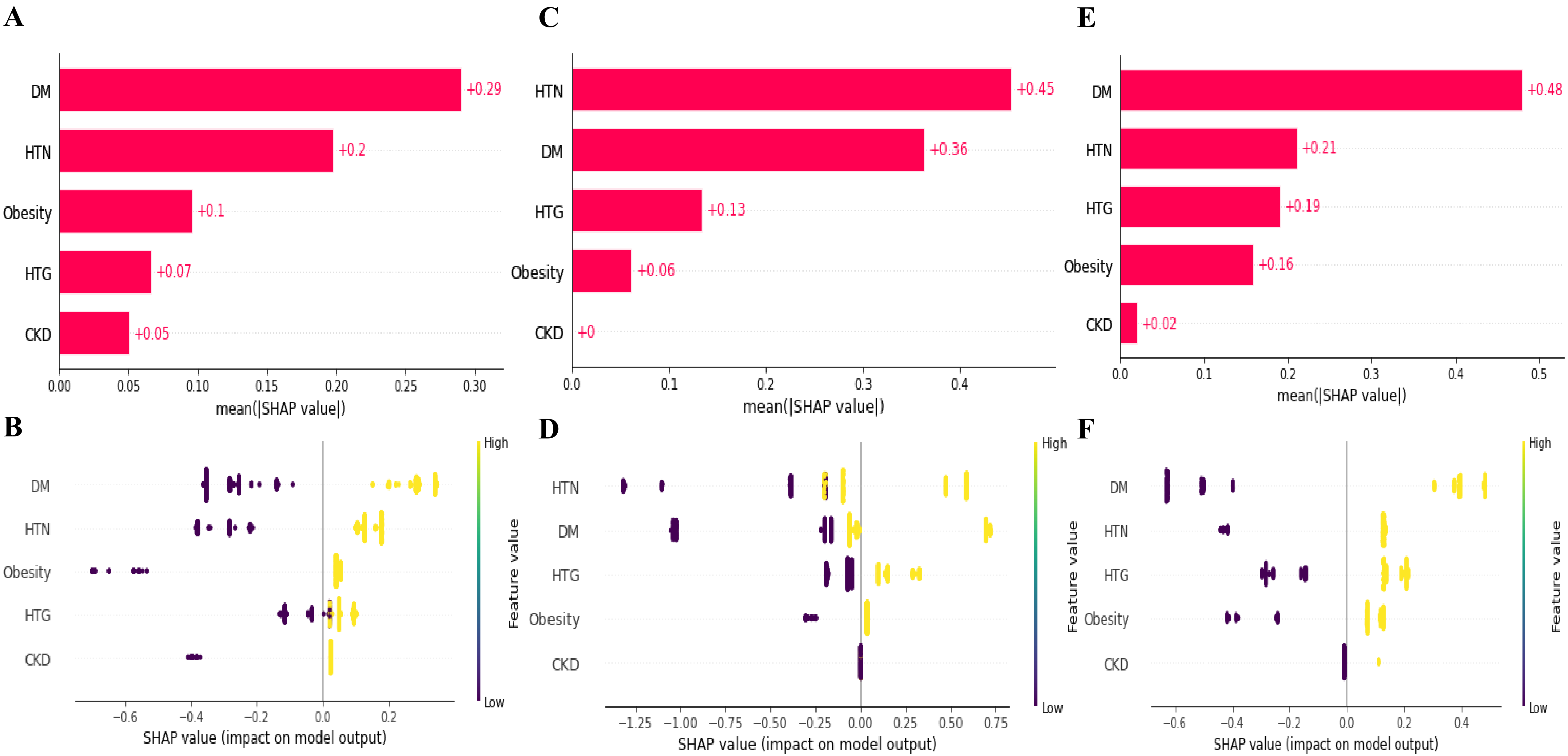

A
DM
HTN
Obesity
HTG
CKD
+0.29
+0.2
+0.1
+0.07
+0.05
0.00
0.05
0.10
0.15
0.20
0.25
0.30
mean(|SHAP value|)
B
DM
HTN
Obesity
HTG
CKD
−0.6
−0.4
−0.2
0.0
0.2
SHAP value (impact on model output)
High
Low
Feature value
C
HTN
DM
HTG
Obesity
CKD
+0.45
+0.36
+0.13
+0.06
+0
0.0
0.1
0.2
0.3
0.4
mean(|SHAP value|)
D
HTN
DM
HTG
Obesity
CKD
−1.25
−1.00
−0.75
−0.50
−0.25
0.00
0.25
0.50
0.75
SHAP value (impact on model output)
High
Low
Feature value
E
DM
HTN
HTG
Obesity
CKD
+0.48
+0.21
+0.19
+0.16
+0.02
0.0
0.1
0.2
0.3
0.4
0.5
mean(|SHAP value|)
F
DM
HTN
HTG
Obesity
CKD
−0.6
−0.4
−0.2
0.0
0.2
0.4
SHAP value (impact on model output)
High
Low
Feature value

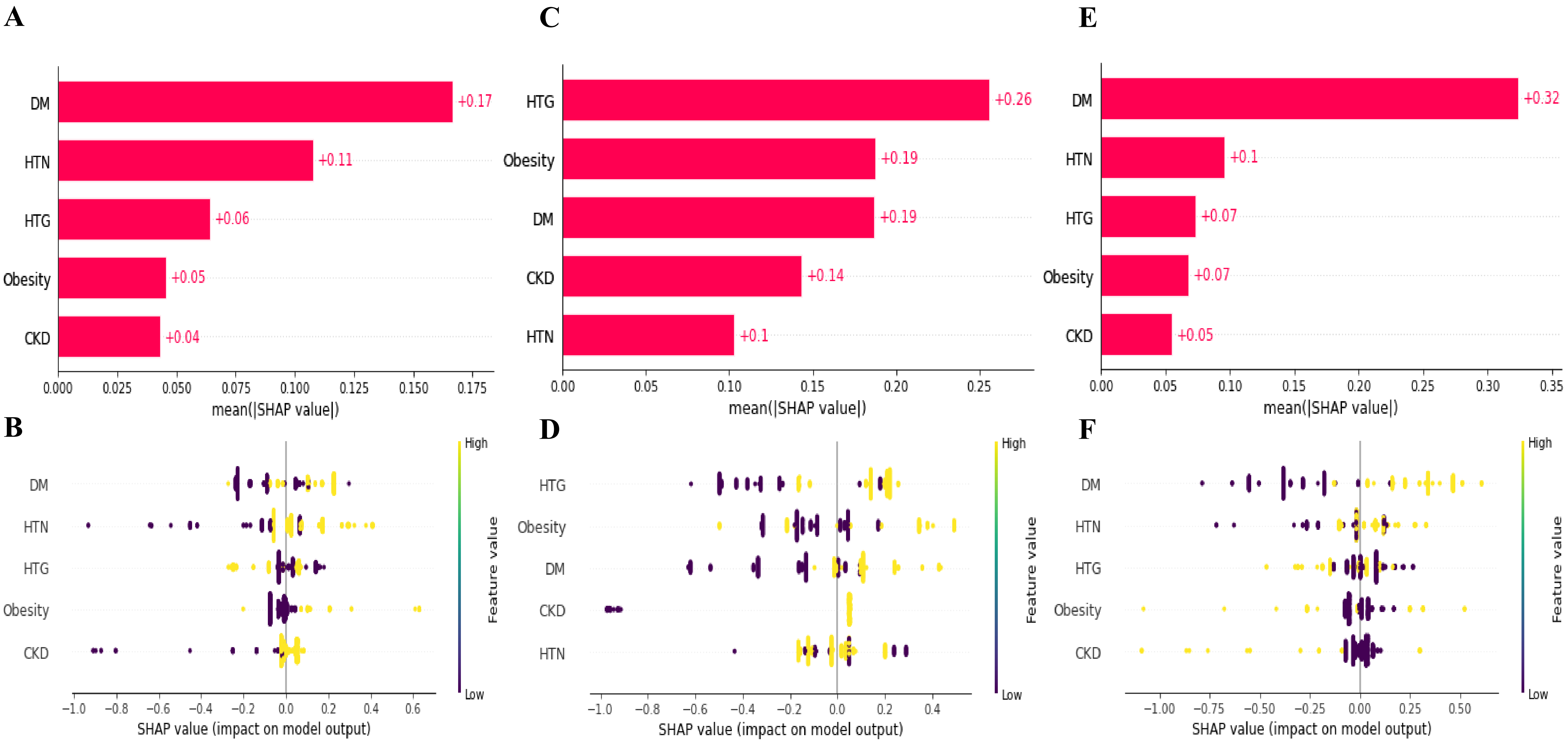
A
DM
HTN
HTG
Obesity
CKD
+0.17
+0.11
+0.06
+0.05
+0.04
0.000 0.025 0.050 0.075 0.100 0.125 0.150 0.175
mean(|SHAP value|)
C
HTG
Obesity
DM
CKD
HTN
+0.26
+0.19
+0.19
+0.14
+0.1
0.00 0.05 0.10 0.15 0.20 0.25
mean(|SHAP value|)
E
DM
HTN
HTG
Obesity
CKD
+0.32
+0.1
+0.07
+0.07
+0.05
0.00 0.05 0.10 0.15 0.20 0.25 0.30 0.35
mean(|SHAP value|)
B
DM
HTN
HTG
Obesity
CKD
High
Low
Feature value
−1.0 −0.8 −0.6 −0.4 −0.2 0.0 0.2 0.4 0.6
SHAP value (impact on model output)
D
HTG
Obesity
DM
CKD
HTN
High
Low
Feature value
−1.0 −0.8 −0.6 −0.4 −0.2 0.0 0.2 0.4
SHAP value (impact on model output)
F
DM
HTN
HTG
Obesity
CKD
High
Low
Feature value
−1.00 −0.75 −0.50 −0.25 0.00 0.25 0.50
SHAP value (impact on model output)
