# Supplementary material for: Machine Learning Analysis of Sex Differences in Cardiovascular-Kidney-Metabolic Risk Factors and Prognosis Among Patients With Moderate-to-Severe Coronary Artery Calcification: Prospective Cohort Study
Source: J Med Internet Res. 2026 Jul 16;28:e82742. doi: 10.2196/82742 (PMC13424754; doi:10.2196/82742)
Supplement: Multimedia Appendix 2 [file jmir_v28i1e82742_app2.docx]

**Supplemental Table**

Supplemental Table 1 Definition of various clinical outcomes

Supplemental Table 2 Comparison of various machine learning models’ performance.

Supplemental Table 3 Hyperparameter of the XGBoost model.

Supplemental Table 4 Baseline characteristics of the CAD participants in the external validation cohort.

| Supplemental Table 1 Definition of various clinical outcomes | |
| --- | --- |
| Outcomes | Definition |
| Major adverse cardiovascular events | the composite of all-cause mortality, non-fatal myocardial infarction, and unplanned repeat revascularization |
| All-cause mortality | The various causes of death accepted by medical institution certification. |
| Non-fatal myocardial infarction | Positive cardiac troponin levels along with typical chest pain, distinctive electrocardiogram abnormalities, angiographic evidence of intracoronary thrombus, or imaging evidence of acute myocardial necrosis or regional wall motion abnormalities |
| Unplanned repeat revascularization | any repeat percutaneous coronary intervention or surgical intervention occurring after hospital discharge excluding scheduled staged percutaneous coronary intervention instances |

| **Supplemental Table 2 Comparison of various machine learning models’ performance** | | | | | |
| --- | --- | --- | --- | --- | --- |
|  | AUC (95% CI) | AP (95% CI) | NPV (95% CI) | PPV (95% CI) | F1 score (95% CI) |
| **train set** |  |  |  |  |  |
| Logistic regression | 0.82 (0.79–0.85) | 0.81 (0.78–0.84) | 0.87 (0.84–0.90) | 0.68 (0.63–0.73) | 0.76 (0.72–0.80) |
| Random forest | 0.85 (0.82–0.88) | 0.84 (0.81–0.87) | 0.89 (0.86–0.92) | 0.72 (0.67–0.77) | 0.79 (0.75–0.83) |
| Support vector machine | 0.93 (0.91–0.95) | 0.93 (0.91–0.95) | 0.94 (0.92–0.96) | 0.85 (0.81–0.89) | 0.89 (0.86–0.92) |
| Extreme Gradient Boosting | 0.96 (0.94–0.98) | 0.96 (0.94–0.98) | 0.97 (0.95–0.99) | 0.92 (0.89–0.95) | 0.94 (0.92–0.96) |
| **test set** |  |  |  |  |  |
| Logistic regression | 0.78 (0.74–0.82) | 0.77 (0.73–0.81) | 0.85 (0.81–0.89) | 0.62 (0.57–0.67) | 0.73 (0.68–0.78) |
| Random forest | 0.80 (0.76–0.84) | 0.80 (0.76–0.84) | 0.88 (0.84–0.92) | 0.65 (0.60–0.70) | 0.74 (0.69–0.79) |
| Support vector machine | 0.90 (0.87–0.93) | 0.92 (0.89–0.95) | 0.92 (0.89–0.95) | 0.78 (0.73–0.83) | 0.84 (0.80–0.88) |
| Extreme Gradient Boosting | 0.92 (0.89–0.95) | 0.92 (0.89–0.95) | 0.93 (0.90–0.96) | 0.80 (0.75–0.85) | 0.86 (0.82–0.90) |
| external validation set |  |  |  |  |  |
| EXtreme Gradient Boosting | 0.86 (0.78–0.94) | 0.71 (0.58–0.84) | 0.91 (0.86–0.96) | 0.72 (0.55–0.89) | 0.68 (0.55–0.81) |
| AUC, Area Under the Curve; AP, Average Precision; NPV, Negative Predictive Value; PPV, Positive Predictive Value. | | | | | |

| **Supplemental Table 3 Hyperparameter of the XGBoost model** | | |
| --- | --- | --- |
| XGBoost | Hyperparameter | Search Range / Grid |
| Objective | binary:logistic | Fixed |
| n_estimators (Number of trees) | 500 | 100–1000 |
| max_depth | 9 | 3–12 |
| learning_rate (eta) | 0.1 | 0.01–0.3 |
| subsample | 0.6 | 0.5–1.0 |
| colsample_bytree | 0.8 | 0.6–1.0 |
| gamma | 0 | 0–5 |
| min_child_weight | 1 | 1–10 |
| reg_alpha | 0 | 0–1 |
| reg_lambda | 1 | 0–2 |
| eval_metric | auc | Fixed |

| Supplemental Table 4. Baseline characteristics of the CAD participants in the external validation cohort | | | | | |
| --- | --- | --- | --- | --- | --- |
|  |  | Overall | MACE | no MACE | p |
| n |  | 127 | 14 | 113 |  |
| Male (%) |  | 89 (70.1) | 9 (64.3) | 80 (70.8) | 0.047 |
| Age,y |  | 64.60 [55.95, 70.25] | 69.55 [67.85, 76.72] | 63.90 [54.50, 69.50] | 0.012 |
| CKM risk factors |  |  |  |  |  |
| Chronic kidney disease, n (%) | | 12 (9.4) | 5 (35.7) | 7 (6.2) | 0.002 |
| Diabetes, n (%) |  | 62 (48.8) | 7 (50.0) | 55 (48.7) | 0.079 |
| Obesity, n (%) |  | 65 (51.2) | 7 (50.0) | 58 (51.3) | 0.068 |
| Hypertension, n (%) |  | 87 (68.5) | 8 (57.1) | 79 (69.9) | 0.506 |
| Hypertriglyceridemia, n (%) | | 94 (74.0) | 10 (71.4) | 84 (74.3) | 0.077 |
| Values are median [interquartile range], or n (%) as appropriate. CAD, coronary artery disease; HDL-C, high-density lipoprotein cholesterol; LDL-C, low-density lipoprotein cholesterol; CKM, cardiovascular-kidney-metabolic; eGFR, estimated glomerular filtration rate. | | | | | |
